# A lipidated TLR7/8 adjuvant enhances the efficacy of a vaccine against fentanyl in mice

**DOI:** 10.1038/s41541-023-00694-y

**Published:** 2023-07-10

**Authors:** Shannon M. Miller, Bethany Crouse, Linda Hicks, Hardik Amin, Shelby Cole, Helene G. Bazin, David J. Burkhart, Marco Pravetoni, Jay T. Evans

**Affiliations:** 1 Department of Biomedical and Pharmaceutical Sciences, Center for Translational Medicine, University of Montana, Missoula, MT USA; 2 Inimmune Corporation, Missoula, MT USA; 3 Department of Pharmacology, University of Minnesota Medical School, Minneapolis, MN USA; 4 Department of Veterinary Population Medicine, University of Minnesota, St. Paul, MN USA; 5 Division of Biological Sciences, University of Montana, Missoula, MT USA; 6 Center for Immunology, University of Minnesota, Minneapolis, MN USA; 7 Department of Psychiatry and Behavioral Sciences, University of Washington School of Medicine, Seattle, WA USA

**Keywords:** Adjuvants, Addiction, Conjugate vaccines, Toll-like receptors, Antibodies

## Abstract

Opioid use disorders (OUD) and opioid-related fatal overdoses are a public health concern in the United States. Approximately 100,000 fatal opioid-related overdoses occurred annually from mid-2020 to the present, the majority of which involved fentanyl or fentanyl analogs. Vaccines have been proposed as a therapeutic and prophylactic strategy to offer selective and long-lasting protection against accidental or deliberate exposure to fentanyl and closely related analogs. To support the development of a clinically viable anti-opioid vaccine suitable for human use, the incorporation of adjuvants will be required to elicit high titers of high-affinity circulating antibodies specific to the target opioid. Here we demonstrate that the addition of a synthetic TLR7/8 agonist, INI-4001, but not a synthetic TLR4 agonist, INI-2002, to a candidate conjugate vaccine consisting of a fentanyl-based hapten, F_1_, conjugated to the diphtheria cross-reactive material (CRM), significantly increased generation of high-affinity F_1_-specific antibody concentrations, and reduced drug distribution to the brain after fentanyl administration in mice.

## Introduction

Opioid use disorders (OUD) and opioid-related fatal overdoses are a growing public health concern in the United States. Over 2.5 million individuals are currently diagnosed with an OUD involving heroin, prescription opioids such as oxycodone and hydrocodone, synthetic opioids such as fentanyl, or multiple compounds. While deaths due to heroin and prescription opioids have remained steady or dropped slightly since 2015, the incidence of deaths due to other synthetic opioids, such as fentanyl, has increased dramatically from roughly 3 deaths per 100,000 in 2015 to 10 deaths per 100,000 in 2018^1,2^. More recently, the SARS-CoV-2 pandemic has exacerbated the opioid crisis with over 100,000 fatal overdoses (approximately 30 deaths per 100,000) occurring over the 12-month period ending in June 2021^3^, the highest number of recorded deaths in a 1-year period, and this trend continued in 2022^3^. While most patients engage in poly-drug misuse or may transition between heroin and other opioids, clinical data indicate that the majority of fatal overdoses involved fentanyl or a fentanyl analog^4,5^.

Current medication-assisted treatment (MAT) to treat OUD consists of opioid receptor agonists, partial agonists, and antagonists, such as methadone and buprenorphine, while the opioid antagonist naloxone is used as an overdose reversal agent (e.g., Narcan®). New approaches to treat and prevent OUD and overdose are urgently needed, in particular with respect to protecting from death due to fentanyl overdose. Success rates associated with MAT are typically less than 50 percent due to limited access (only 1 in 5 patients access MAT), patient compliance, social stigma regarding OUD, and regulatory hurdles^6^. For example, the opioid receptor antagonist naltrexone requires detoxification from opioid use prior to initiating therapy in order to avoid withdrawal symptoms and interferes with the use of opioid analgesics for pain management. The opioid receptor agonist methadone and the partial agonist buprenorphine have abuse and diversion liabilities of their own, and often must be administered daily in a healthcare facility or an in-patient addiction clinic, limiting patient access and compliance. During the ongoing SARS-CoV-2 pandemic patients with OUD were further limited in their access to MAT, and therefore their treatment was further complicated, often pushing people to seek illicit sources of methadone in self-medication and harm reduction attempts. FDA-approved medications for the rescue of overdose, mainly consisting of various formulations of the opioid antagonist naloxone, have limited efficacy against fentanyl poisoning because of the relatively shorter half-life and limited potency of naloxone compared to fentanyl. These data suggest that the development of novel treatments targeting fentanyl, fentanyl analogs, or illicit street mixtures containing fentanyl (e.g., heroin/fentanyl) may reduce the incidence of fatal overdoses in patients with OUD that are currently using opioids or that are at risk of relapse and overdose, patients with a substance use disorder (SUD) that may accidentally be exposed to fentanyl (e.g., cocaine laced with fentanyl) and other populations that may be at risk of accidental or deliberate exposure to fentanyl.

The use of vaccines as a complementary strategy to treat OUD and prevent fatal overdoses has shown great promise in preclinical studies involving various haptens, carrier proteins, adjuvants, challenge drugs, and animal models (reviewed in ref. ^6^). Vaccines specifically targeted to fentanyl have been evaluated preclinically in mice, rats, and nonhuman primates^7–14^. In particular, we recently reported a series of conjugate vaccines containing haptens targeting fentanyl and selected analogs^9,15,16^. A fentanyl-based (F_1_) hapten conjugated to CRM_197_ (F_1_-CRM) was chosen as a lead vaccine candidate that warranted further evaluation and development^9^. Vaccination with F_1_-CRM plus aluminum hydroxide (alum) adjuvant protected rodents against fentanyl-induced antinociception, respiratory depression, and bradycardia, and significantly decreased the concentration of opioids in the brain after subcutaneous (SC) fentanyl and sufentanil challenges in mice and rats^9^. F_1_-CRM was also effective against acetylfentanyl^15^. This F_1_-CRM vaccine was also effective at reducing fentanyl intravenous (IV) self-administration in rats^9^. Importantly, vaccination with F_1_-CRM did not interfere with commonly used injectable or volatile anesthetics, nor methadone, oxycodone, or the reversal of their pharmacological effects by naloxone in rats^9^.

The development of an effective anti-opioid vaccine in humans will benefit from vaccine technologies that result in high titers of circulating opioid-specific antibodies with high affinity to fentanyl and closely related analogs. Previous human clinical trials using vaccines for nicotine or cocaine demonstrated that only about 30% of immunized individuals, those with the highest drug-specific antibody titers, were protected^17,18^. Therefore, improved adjuvants and delivery technologies are likely necessary to enhance clinical outcomes for vaccines targeting OUD. A variety of adjuvants have been evaluated in vaccines targeting opioids or other drugs of abuse. A number of groups have reported that the use of alum increased anti-opioid antibody titers^6,8,19–22^. Recent advancements in the development and clinical testing of pattern recognition receptor (PRR) ligands as vaccine adjuvants have opened the door to new safe, and effective adjuvant systems. Several of these adjuvants have demonstrated success in anti-opioid vaccines^19,23,24^, while others have shown mixed results^25,20^ (reviewed in ref. ^6^). TLR7/8 adjuvants may be particularly well suited for opioid vaccines due to the potentially enhanced effect of IgG2a antibodies in protecting mice from opioid challenge^26^ and the ability of TLR7/8 agonists to bias a Th1 immune response and enhance antigen-specific IgG2a titers^27–29^. We, therefore, tested the lead anti-fentanyl vaccine candidate, F_1_-CRM, in combination with synthetic TLR7/8 (INI-4001) and TLR4 (INI-2002) adjuvants. We found that the combination of INI-4001 and alum (Alhydrogel) significantly increased F_1_-specific IgG antibody concentrations, while the addition of INI-2002 did not improve antibody concentration. Further, the use of INI-4001 with alum increased F_1_-specific antibody avidity and enhanced protection against fentanyl challenge in mice. We extended these findings in a rat model of fentanyl challenge, overdose, and self-administration and a newly developed mini-pig model of fentanyl challenge and overdose (Crouse et al.^30^, NPJ Vaccines, concurrent dual submission under review). In all three animal models, the INI-4001 + alum adjuvanted F_1_-CRM vaccine increased F_1_ specific antibody titers and improved efficacy against fentanyl challenge compared to F_1_-CRM + alum and unadjuvanted F_1_-CRM controls. These data indicate that the INI-4001 adjuvanted F_1_-CRM fentanyl vaccine described here may be an effective vaccine against OUD and opioid-related fatal overdoses.

## Results

### INI-4001 is a synthetic TLR7/8 agonist

**Fig. 1:**
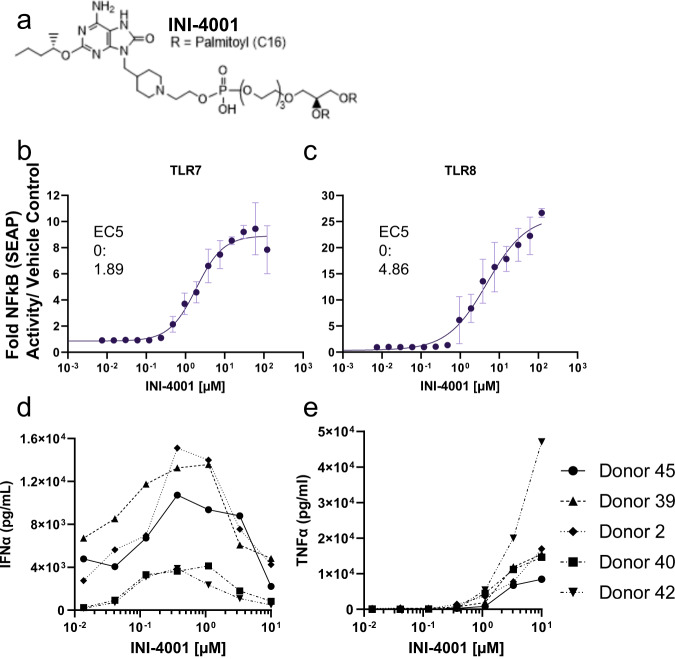
INI-4001 is a synthetic TLR7/8 agonist. **a** Chemical structure of INI-4001. **b**, **c** SEAP activation in HEK293 cells expressing human TLR7 (**b**) or human TLR8 (**c**) by indicated doses of INI-4001. Data were reported as fold change in SEAP production over vehicle control and shown as mean ± SEM. **d**, **e** Human PBMCs from five different donors were stimulated with indicated doses of INI-4001 for 24 h. Supernatants were then analyzed for IFNα (**d**) and TNFα (**e**) production by ELISA.

INI-4001 (Fig. 1a) was confirmed as a TLR7/8 agonist using human HEK TLR7 and human HEK TLR8 reporter cells, as previously described in ref. ^27^. We found that INI-4001 induced signaling and SEAP production via both human TLR7 (Fig. 1b) and TLR8 (Fig. 1c). INI-4001 is ~2.6-fold more potent via TLR7 compared to TLR8 (Fig. 1b; EC50 TLR7 = 1.89, EC50 TLR8 = 4.86). Further, INI-4001 failed to stimulate activity in human HEK TLR4 cells (Supplementary Fig. 1). To confirm the activity of INI-4001, we stimulated freshly isolated human PBMCs with INI-4001 for 24 h, at which point cell supernatants were analyzed for IFNα and TNFα (Fig. 1d, e). In humans, IFNα is almost exclusively produced via TLR7 stimulation in pDCs, while TNFα is preferentially produced via TLR8 stimulation^31,32^. The increased potency of INI-4001 in activating TLR7 compared to TLR8 in HEK cells is mirrored in IFNα vs TNFα production in hPBMCs where lower concentrations of compound elicit IFNα responses (Fig. 1d) compared to the concentration of INI-4001 required to elicit TNFα production (Fig. 1e). Taken together, these data demonstrate that INI-4001 signals through both human TLR7 and TLR8 with a bias toward TLR7.

### INI-2002 is a synthetic TLR4 agonist

**Fig. 2:**
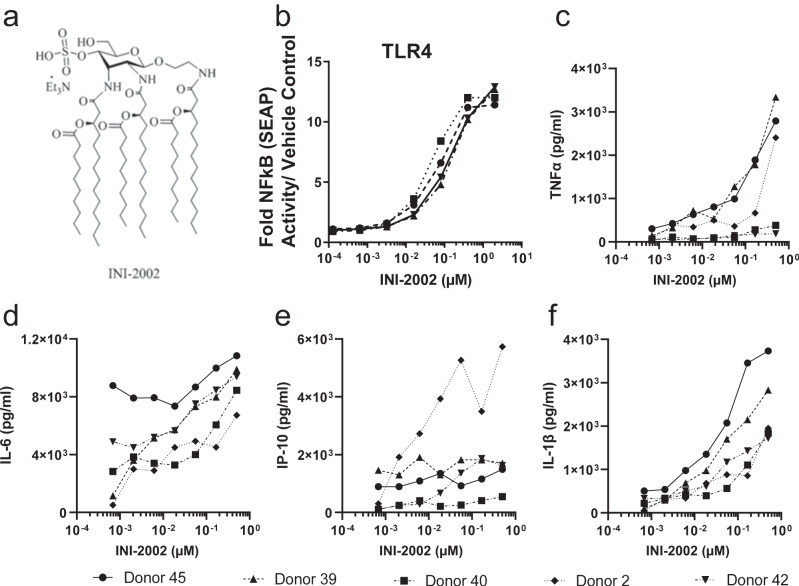
INI-2002 is a synthetic TLR4 agonist. **a** Chemical structure of INI-2002. **b** SEAP activation in HEK293 cells expressing human TLR4 by indicated doses of INI-2002. Data were reported as fold changes in SEAP production over vehicle control. **c**–**f** Human PBMCs from five different donors were stimulated with indicated doses of INI-2002 for 24 h. Supernatants were then analyzed for indicated cytokine production using a custom U-PLEX MesoScale Discovery (MSD) assay for all cytokines shown.

INI-2002 (Fig. 2a) was confirmed as a TLR4 agonist using human HEK TLR4 reporter cells (Fig. 2b) using the same methods as for the human HEK TLR7 and HEK TLR8 reporter cells. INI-2002 demonstrated dose-responsive activation of NF-kB, as measured by SEAP production, in the human TLR4 HEK reporter cells (Fig. 2b). Additionally, INI-2002 failed to stimulate activity in human HEK TLR7 and human HEK TLR8 cells (Supplementary Fig. 1). Further, in hPBMCs, we found that INI-2002 signals through both the TRIF- and MyD88-dependent TLR4 signaling pathways based on the production of TNFα and IL-6 (MyD88-dependent; Fig. 2c, d) and IP-10/CXCL10 (TRIF-dependent; Fig. 2e). IL-1β (Fig. 2f) also may be produced via a TRIF-dependent alternative pathway in monocytes after TLR4 stimulation^33^.

### INI-4001 (TLR7/8 ligand), but not INI-2002 (TLR4 ligand), in combination with alum and F_1_-CRM significantly increased anti-F_1_ antibody titers and biased an IgG2a response

**Fig. 3:**
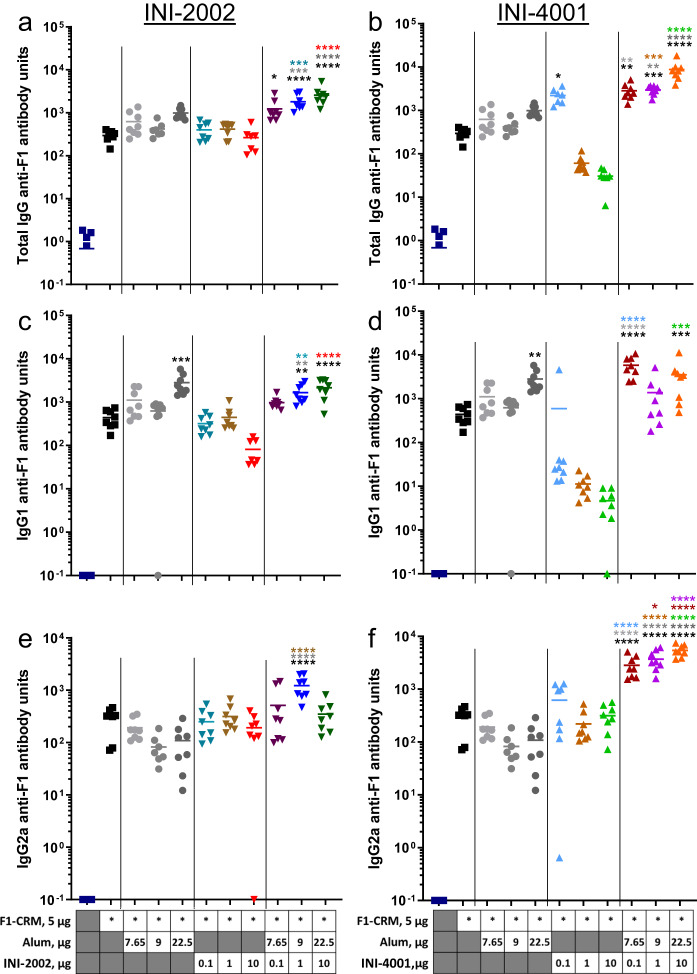
Adjuvanting F_1_-CRM with INI-4001 plus alum preferentially increases anti-F_1_ IgG2a antibody concentration. Mice were vaccinated twice, IM, with 5 µg F_1_-CRM plus 7.68, 9, or 22.5 µg alum, 0.1, 1, or 10 µg INI-2002, 0.1, 1, or 10 µg INI-4001, the combination of INI-2002 + alum, or the combination of INI-4001 + alum as indicated. Fourteen days after the second vaccination, blood was collected, and anti-F_1_ IgG (**a**, **b**), IgG1 (**c**, **d**), and IgG2a (**e**, **f**) antibody concentrations were measured by ELISA. Antibody responses to vaccines adjuvanted with INI-2002 are shown on the left (**a**, **c**, **e**). Antibody responses to vaccines adjuvanted with INI-4001 are shown on the right (**b**, **d**, **f**). Statistical analysis was conducted by one-way ANOVA with Fisher’s LSD for multiple comparisons (GraphPad Prism). *p ≤ 0.05, **p ≤ 0.01, ***p ≤ 0.001, ****p ≤ 0.0001; color of asterisks indicates comparison group.

Previously published data demonstrated that F_1_-CRM combined with alum elicited high-affinity anti-F_1_ serum IgG antibody titers that bound fentanyl and protected mice and rats against fentanyl-induced effects after challenge^7,9^. Here, we investigated whether the inclusion of either a synthetic TLR7/8 or TLR4 adjuvant would significantly increase anti-F_1_ antibody titers when compared to F_1_-CRM plus alum, and/or allow for a substantial reduction in the amount of F_1_ -CRM antigen required to achieve high titers (antigen dose sparing). In particular, we were also interested in determining the levels of anti-F_1_ IgG2a antibodies, as these have been reported to correlate with enhanced protection against opioid challenge^26,30^. Mice were injected intramuscularly (IM) twice, at days 0 and 14, with F_1_-CRM, F_1_-CRM + alum, F_1_-CRM + alum + INI-2002, or F_1_-CRM + alum + INI-4001 as denoted in Fig. 3. A fixed dose of 5 µg F_1_-CRM was used for each vaccination, ~10-fold less than previously reported^9^. INI-2002 and INI-4001 adjuvants were evaluated at 0.1, 1, and 10 µg per injection. The amount of alum (Alhydrogel®) was fixed at 1.5x the total mass of F_1_-CRM plus adjuvant, the amount of Alhydrogel® required for complete adsorption of adjuvant and ~80–90% of F_1_-CRM (Supplementary Fig. 2). After resting the mice for 14 days following the second injection, blood was collected for analysis of anti-F_1_ IgG, IgG1, and IgG2a antibody concentrations in serum (Fig. 3). Anti-F_1_ IgG antibody concentrations were significantly increased compared to F_1_-CRM alone by the addition of alum + INI-2002 (Fig. 3a), 0.1 µg INI-4001 alone, or alum + INI-4001 (Fig. 3b). Anti-F_1_ IgG1 antibody concentrations were significantly increased compared to F_1_-CRM alone when F_1_-CRM was combined with 22.5 µg alum (Fig. 3c, d), 9 µg alum + 1 µg INI-2002, 22.5 µg alum + 10 µg INI-2002 (Fig. 3c), 7.65 µg alum + 0.1 µg INI-4001, or 22.5 µg alum + 10 µg INI-4001 (Fig. 3d). However, neither 22.5 µg alum + 10 µg INI-2002 nor 22.5 µg alum + 10 µg INI-4001 resulted in anti-F_1_ IgG1 concentrations that were significantly higher than 22.5 µg alum alone (Fig. 3c and d, respectively). This indicates that the significantly increased anti-F_1_ IgG1 was driven by alum rather than the combination of alum with INI-2002 or INI-4001. Of note, when F_1_-CRM was adjuvanted with INI-4001 alone (no alum), the anti-F_1_ IgG1 antibody levels were significantly lower than antigen alone and decreased with increasing adjuvant concentration (Fig. 3d). These data suggest a very strong Th1-polarizing adjuvant effect of INI-4001 in the absence of alum. Anti-F_1_ IgG2a antibody concentrations were significantly increased over F_1_-CRM by using a combination of 9 µg alum + 1 µg INI-2002 (Fig. 3e) or all doses of alum+INI-4001 (Fig. 3f). Of note, no group vaccinated with F_1_-CRM with a single adjuvant elicited significantly increased anti-F_1_ IgG2a titers compared to F_1_-CRM. The groups vaccinated with combination adjuvants that did elicit significantly increased anti-F_1_ IgG2a titers compared to F_1_-CRM also drove significantly increased anti-F_1_ IgG2a titers compared to F_1_-CRM adjuvanted with dose-matched single adjuvant controls as denoted by color-coded asterisks in Fig. 3e, f. The combination of alum + INI-4001 elicited a significant, dose-responsive increase in anti-F_1_ IgG2a antibody titers (Fig. 3f). While concentrations of anti-F_1_ IgG and IgG1 antibodies were similar in groups containing alum+INI-2002 compared to alum + INI-4001, anti-F_1_ IgG2a antibody concentrations consistently trended higher in groups adjuvanted with alum+INI-4001 compared to alum + INI-2002 (Fig. 3a vs b, c vs d, and e vs f). These data, taken together, indicate that the use of alum+INI-4001 as a combination adjuvant preferentially increased F_1_-specific IgG2a antibody concentrations compared to F_1_-CRM, F_1_-CRM + alum, and F_1_-CRM + INI-4001.

**Fig. 4:**
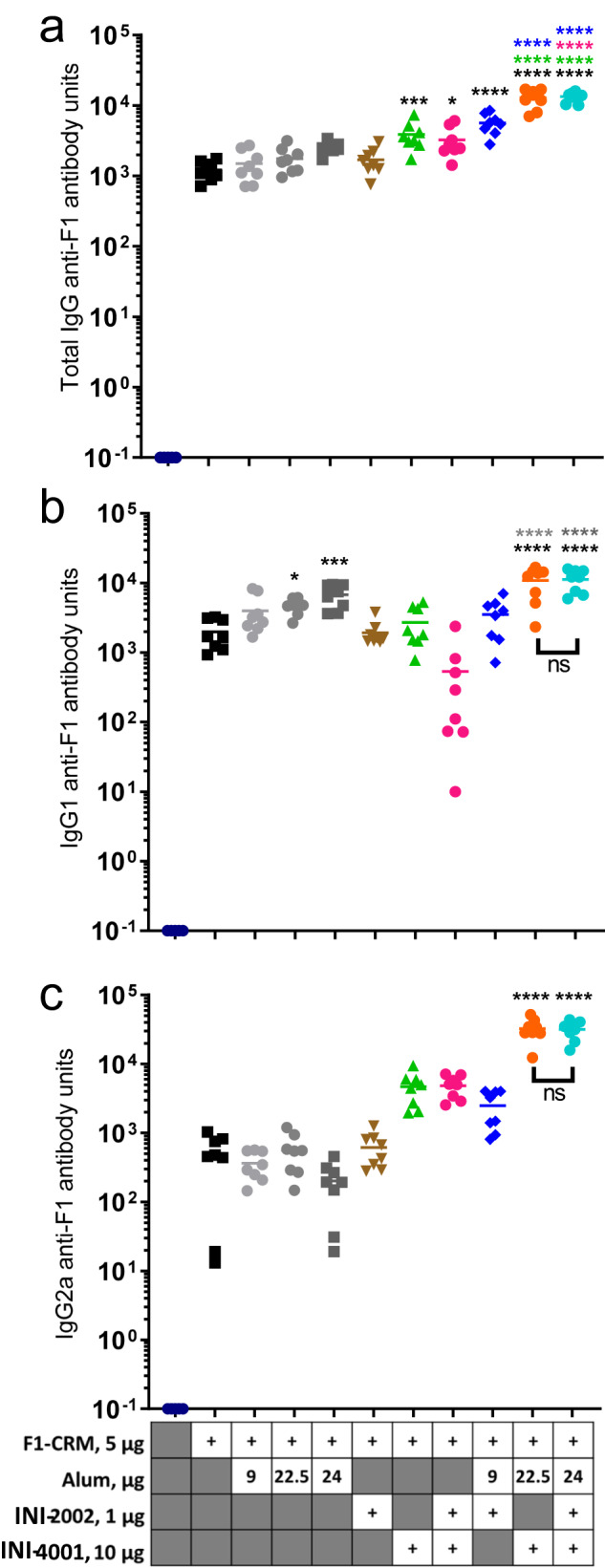
The addition of INI-2002 to INI-4001 plus alum does not further increase F1-specific antibody concentrations. Mice were vaccinated twice, IM, with 5 µg F1-CRM plus 9, 22.5, or 24 µg alum, 1 µg INI-2002, 10 µg INI-4001, the combination of INI-2002 + INI-4001, INI-2002 + alum, INI-4001 + alum, or INI-2002 + INI-4001 + alum as indicated. Fourteen days after the second vaccination, blood was collected and anti-F_1_ IgG (**a**), IgG1 (**b**), and IgG2a (**c**) antibody concentrations were measured by ELISA. Statistical analysis was conducted by one-way ANOVA with Fisher’s LSD for multiple comparisons (GraphPad Prism). *p ≤ 0.05, **p ≤ 0.01, ***p ≤ 0.001, ****p ≤ 0.0001; color of asterisks indicates comparison group.

We next investigated whether the adjuvant combination of INI-4001 + INI-2002 (with or without alum) could further increase antibody titers compared to single adjuvant controls. TLR4 and TLR7/8 agonists have been reported to synergize to produce increased antibody titers in the context of infectious disease vaccines^28,29^. As we were particularly interested in increasing anti-F_1_ IgG2a antibody titers, we moved forward with 1 µg INI-2002 and 10 µg INI-4001 as these doses of TLR4 and TLR7/8 adjuvant, respectively, when combined with alum elicited the greatest increase in anti-F_1_ IgG2a antibody concentrations compared to controls (Fig. 3e, f). Mice were vaccinated twice, IM, on days 0 and 14 with 5 µg F_1_-CRM alone or with alum, 1 µg INI-2002, 10 µg INI-4001, alum + INI-2002, alum + INI-4001, or all three adjuvants in combination (Fig. 4). After resting the mice for 14 days following the second injection, blood was collected and anti-F_1_ IgG, IgG1, and IgG2a antibody concentrations were measured in serum. Anti-F_1_ IgG concentrations were significantly increased compared to F_1_-CRM alone when INI-4001, INI-4001 + INI-2002, alum + INI-2002, alum + INI-4001, or the combination of all three adjuvants was used (Fig. 4a). Significantly higher anti-F_1_ IgG antibody concentrations were elicited by combination adjuvants alum + INI-4001 or alum + INI-2002 + INI-4001 compared to single adjuvant controls (Fig. 4a), although the triple adjuvant combination did not elicit significantly higher antibody concentrations compared to alum+INI-4001. Anti-F_1_ IgG1 antibody concentrations were significantly increased compared to F_1_-CRM alone when F_1_-CRM was adjuvanted with 9 or 22.5 µg alum, alum + INI-4001, or alum + INI-2002 + INI-4001 (Fig. 4b). The triple combination adjuvant did not further increase antibody concentrations compared to alum + INI-4001 (Fig. 4b). Anti-F_1_ IgG1 concentrations elicited by both alum + INI-4001 and the triple combination adjuvant were also significantly increased compared to single adjuvant controls (Fig. 4b). IgG2a F_1_-specific antibody concentrations were significantly increased compared to F_1_-CRM alone only by the addition of alum + INI-4001 or the triple adjuvant combination (Fig. 4c). Both alum + INI-4001 and the triple adjuvant combination significantly increased anti-F_1_ IgG2a antibody concentrations compared to their single adjuvant controls (Fig. 4c). However, as was found in both anti-F_1_ IgG and IgG1 concentrations, the addition of alum+INI-2002 + INI-4001 did not increase anti-F_1_ IgG2a concentrations compared to alum+INI-4001 (Fig. 4c). Collectively, these data demonstrate that alum + INI-4001 elicited the highest anti- F_1_ IgG, IgG1, and IgG2a concentrations; the presence or absence of INI-2002 in combination with alum + INI-4001 had no effect on the anti-F_1_ antibody concentrations in this study, indicating no synergistic effect of combining both TLR4 and TLR7/8 agonists in this case. On average, F_1_-CRM+alum+INI-4001 increased anti-F_1_ IgG1 concentrations 5.46-fold compared to F_1_-CRM alone and 2.34-fold compared to F_1_-CRM+alum while anti-F_1_ IgG2a concentrations were increased 64.9-fold compared to F_1_-CRM alone and 57.6-fold compared to F_1_-CRM + alum. This demonstrates that the addition of INI-4001 polarized the F_1_-specific immune response more strongly towards a Th1-type immune response compared to a Th2-type immune response. This is further supported by F1-CRM-induced T-cell cytokine secretion, where the addition of INI-4001 significantly increased IFNγ secretion (Supplementary Fig. 3A) but not IL-5 secretion (Supplementary Fig. 3B). Additionally, CRM-specific antibody titers were similarly increased in a Th1-biased manner by using the combination of alum + INI-4001 as adjuvants (Supplementary Fig. 4C). While INI-4001 is a PEGylated compound, differences in the amount of PEG-specific antibodies were not detected across groups, there is some individual variation among mice (Supplementary Fig. 5). However, the presence of PEG-specific antibodies in some mice did not interfere with the ability of a secondary vaccination to increase F_1_-specific antibody concentrations.

### F_1_-CRM interferes with TLR4 activation by INI-2002

**Fig. 5:**
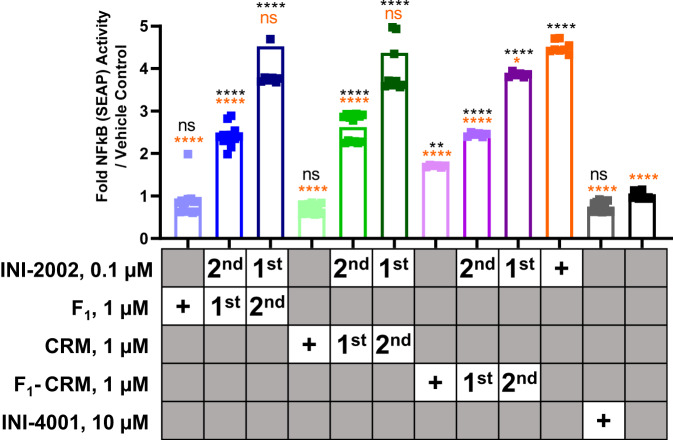
The activation of TLR4 by INI-2002 is blocked by the addition of F_1_, CRM, or F_1_-CRM. HEK cells expressing human TLR4 were stimulated with F_1_, CRM, F_1_-CRM, INI-2002, or the combination of F_1_, CRM, or F_1_-CRM plus INI-2002. The order of addition of F_1_, CRM, or F_1_-CRM and INI-2002 varied when both were used as indicated in the table by either “first” or “second”. The addition of the second component occurred 1 h after the addition of the first component. INI-4001 and vehicle only were included as negative controls. Statistical analysis was conducted by one-way ANOVA with Fisher’s LSD for multiple comparisons (GraphPad Prism). **p ≤ 0.01 and ****p ≤ 0.0001. Orange asterisks and text indicate a comparison to INI-2002, and black asterisks and text indicate a comparison to vehicle only.

Previous reports have provided mixed data on the efficacy of TLR4 agonists as adjuvants for anti-opioid vaccines^20,25^. Some opioids, such as morphine, are reported to be TLR4 agonists^34,35^ while others, including fentanyl, can have TLR4 inhibitory properties in some contexts^36,37^. The TLR4 agonist MPLA did not increase the efficacy of an oxycodone conjugate vaccine, and vaccination of TLR4 deficient mice induced significantly reduced antibodies against oxycodone compared to wild-type mice^20^, further supporting the need for exploring any interaction between TLR4 agonists and components of opioid vaccines. Here we demonstrated minimal efficacy using a synthetic TLR4 ligand (INI-2002). Further, we explored whether F_1_-CRM was capable of signaling through TLR4 or potentially inhibiting the activity of INI-2002. We tested the TLR4 activity of F_1_-CRM as well as F_1_ and CRM separately, using HEK293 cells engineered to express TLR4 and drive an NF-kB dependent reporter gene leading to the production of SEAP. We found that 1 µM F_1_-CRM elicited a modest, but statistically significant, increase in TLR4 activation compared to vehicle control (Fig. 5, light purple vs black). TLR4 activation elicited by 1 µM F_1_-CRM was significantly less than the activation of TLR4 elicited by 0.1 µM INI-2002 (Fig. 5, light purple vs orange). Interestingly, neither 1 µM F_1_ nor 1 µM CRM alone was capable of TLR4 activation (Fig. 5, light blue and light green, respectively, vs black). When 1 µM F_1_-CRM, F_1_, or CRM was added to HEK TLR4 reporter cells for 1 h before the addition of 0.1 µM INI-2002, in all cases, the ability of INI-2002 to activate TLR4 was significantly decreased compared to 0.1 µM INI-2002 alone (Fig. 5, medium blue, green, and purple, respectively, vs orange). If INI-2002 was added to HEK TLR4 cells first, the addition of F_1_ or CRM after 1 h did not change the ability of INI-2002 to activate TLR4 (Fig. 5, dark blue and dark green, respectively, vs orange). However, when INI-2002 was added to HEK TLR4 cells first, followed by F_1_-CRM 1 h later, a modest but statistically significant decrease in TLR4 activation was measured (Fig. 5, dark purple vs orange). While we cannot definitively state that F_1_ inhibited TLR4 activation by INI-2002 due to its ability to bind TLR4 as CRM also inhibited TLR4 activation by INI-2002, the use of a TLR4-based adjuvant for anti-opioid vaccines should nevertheless be carefully assessed given these results and the previously reported interactions between opioids and TLR4.

### INI-4001 significantly increased antibody avidity to F_1_

**Fig. 6:**
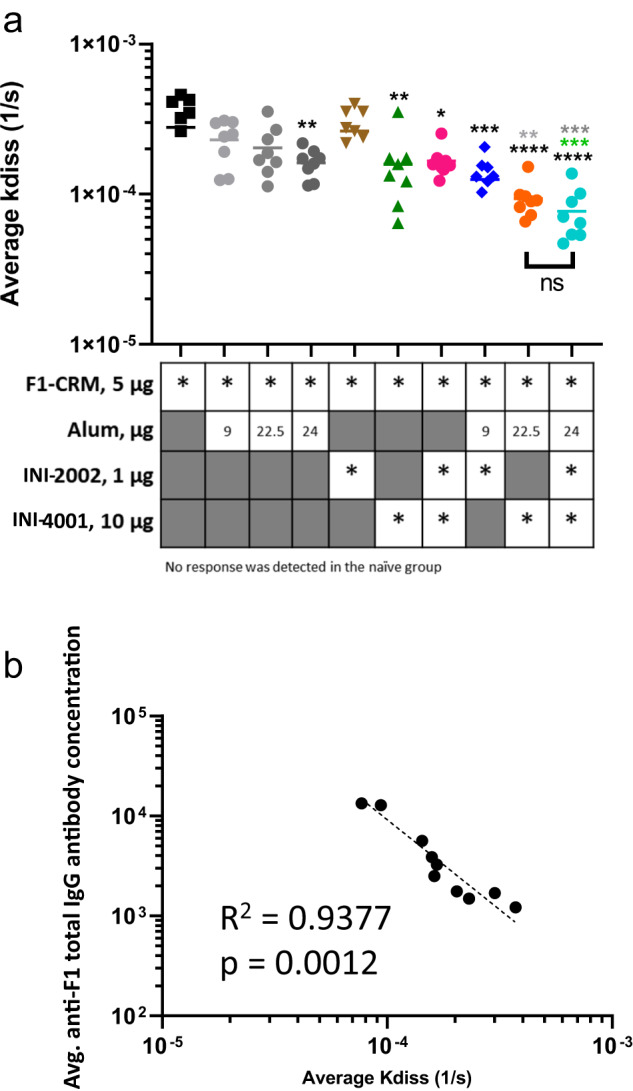
Addition of INI-4001 increases antibody avidity to F_1_. **a** Mouse serum samples from Fig. 3 were used to measure the dissociation rate (*K*_diss_) between F_1_ and polyclonal mouse serum for each individual serum sample using the Octet Red 96e instrument (Fortebio). Dissociation rate constants (*K*_diss_) were calculated by processing raw data using ForteBio HT analysis software version 11.1.3.50. All data were inspected for quality of fit to the calculated curve (*R*^2^ > 0.95), the response between 0.25–3 (nm shift), and residual value <10% of the maximum response fitted to the curve. **b** Average *K*_diss_ per group from (**a**) was plotted against average anti-F_1_ IgG antibody concentration as shown in Fig. 3a. Correlation was calculated via nonlinear regression analysis where both axes are graphed on a log scale. Statistical analysis was conducted by one-way ANOVA with Fisher’s LSD for multiple comparisons (GraphPad Prism). *p ≤ 0.05, **p ≤ 0.01, ***p ≤ 0.001, ****p ≤ 0.0001; color of asterisks indicates comparison group.

We further investigated whether adjuvants, particularly INI-4001, when used alone or in combination with alum, could increase antibody avidity for F_1_ in addition to increasing anti-F_1_ antibody concentrations. We elected to measure affinity against F_1_ instead of fentanyl as we were interested in whether the adjuvants used here increased antibody affinity to the hapten. Using the OctetRed biolayer interferometry (BLI) system, we bound F_1_-biotin to the sensor tip and calculated the average *k*_diss_ (or *k*_off_) of serum F_1_-specific antibodies from individual F_1_-CRM vaccinated mice with or without adjuvants as described above and shown in Fig. 3. Lower values for *k*_diss_ indicate that the F_1_-specific antibodies in the serum sample took longer to dissociate from F_1_ and thus have greater avidity. Representative sensorgrams are shown in Supplementary Fig. 6. Using this method, we found that adjuvant F_1_-CRM with 24 µg alum, INI-4001, INI-4001 + INI-2002, alum + INI-2002, alum + INI-4001, or alum + INI-2002 + INI-4001 significantly decreased the *k*_diss_ compared to F_1_-CRM alone (Fig. 6a). However, only alum + INI-4001 and the triple adjuvant combination significantly decreased *k*_diss_ compared to dose-matched alum adjuvanted controls (Fig. 6a). Consistent with antibody concentration data, the triple adjuvant combination did not further decrease *k*_diss_ compared to alum + INI-4001. We also noted a significant, strong negative linear relationship between average *k*_diss_ and IgG F_1_-specific antibody concentrations in vaccinated mice (Fig. 6b). These data suggest that these adjuvants increased F_1_-specific IgG antibody concentrations and simultaneously drove an enhanced average antibody avidity for F_1_. Here, the increase in both F_1_-specific antibody concentration and avidity is mainly due to the combination adjuvant alum+INI-4001. Given the measured increase in anti-F_1_ antibody concentrations and antibody avidity, we hypothesized that F_1_-CRM+alum+INI-4001 would significantly outperform F_1_-CRM and F_1_-CRM + alum in a fentanyl challenge experiment.

### INI-4001 adjuvanted F_1_-CRM provides superior protection from fentanyl challenge

**Fig. 7:**
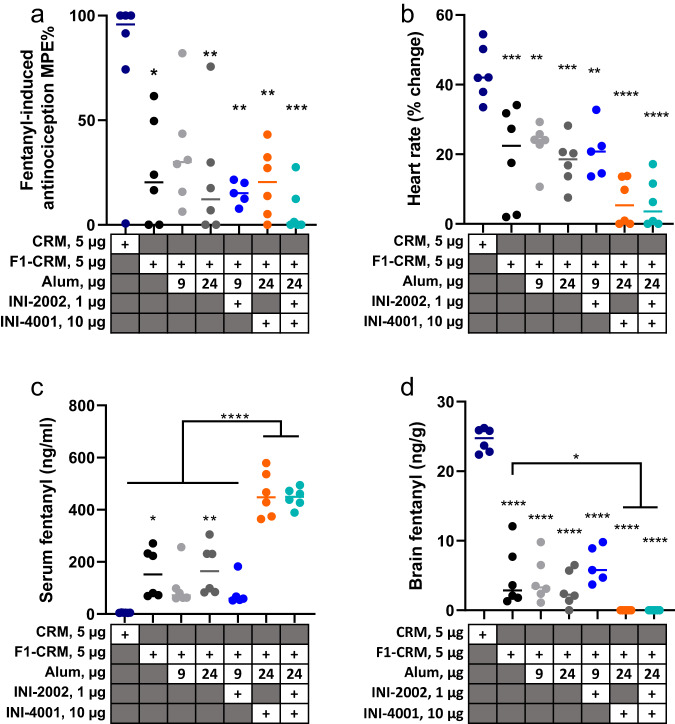
INI-4001 plus alum increases the efficacy of F_1_-CRM to protect mice against fentanyl challenge. Mice were immunized, IM, on days 0, 14, and 28, with indicated vaccine components, followed by drug challenge with 0.05 mg/kg fentanyl s.c. on day 35. **a** Fentanyl-induced antinociception on a hotplate, measured as percent maximum possible effect (%MPE), **b** fentanyl-induced bradycardia, measured as heart rate percent change from baseline, **c** serum fentanyl concentration, and **d** brain fentanyl concentration. Data were mean ± SEM. Sample size: *n* = 6 per group. Statistical analysis was conducted by one-way ANOVA with Tukey’s multiple comparisons post hoc test (GraphPad Prism). *p ≤ 0.05, **p ≤ 0.01, ***p ≤ 0.001, ****p ≤ 0.0001 compared to CRM-only vaccinated control or as noted by bars.

As noted above, alum + INI-4001 significantly increased F_1_-specific antibody concentrations and affinity after vaccination compared to F_1_-CRM alone or single adjuvant controls. We then extended these findings in a murine vaccination and fentanyl challenge model to determine whether the alum+INI-4001 adjuvanted fentanyl vaccine elicited greater protection against fentanyl-induced pharmacological effects and distribution of fentanyl after fentanyl administration. INI-2002 (TLR4 agonist) adjuvanted F_1_-CRM was also included to confirm the previous findings of low adjuvant activity. Mice were vaccinated IM with CRM carrier protein, F_1_-CRM, F_1_-CRM + alum, F_1_-CRM + alum+INI-2002, F_1_-CRM + alum+INI-4001, or F_1_-CRM in combination with all three adjuvants at doses noted in Fig. 7. All mice were challenged with 0.05 mg/kg fentanyl after three vaccinations (Fig. 7a–d). Prior to the challenge, mice were bled, and F_1_-specific antibody titers were measured in serum (Supplementary Fig. 7), where trends were consistent with data reported in Fig. 4. Anti-nociception in the hotplate assay and bradycardia monitored by pulse oximetry were measured as indicators of vaccine efficacy in blocking responses to fentanyl as previously described in refs. ^9,15,16^. Additionally, serum and brain concentrations of fentanyl were measured 30 min post-fentanyl challenge to determine the ability of vaccine-induced antibodies to prevent fentanyl from crossing the blood–brain barrier. After three vaccinations, all mice vaccinated with an F_1_-containing vaccine, except F_1_-CRM + 9 ug alum, demonstrated significantly reduced fentanyl-induced antinociception compared to CRM-only vaccinated mice (Fig. 7a). Further, all mice vaccinated with an F_1_-containing vaccine demonstrated significantly reduced bradycardia (Fig. 7b) compared to CRM-only vaccinated mice. The addition of INI-4001 to F_1_-CRM+alum further reduced bradycardia compared to F_1_-CRM alone, as did the triple adjuvant combination, although this further reduction in bradycardia of F_1_-CRM + alum + INI-4001 compared to F_1_-CRM + alum 24 ug was not statistically significant (Fig. 7b). These data demonstrate that while three vaccinations with 5 µg F_1_-CRM alone is sufficient to protect against fentanyl-induced antinociception, the addition of alum+INI-4001 further increases protection against bradycardia, although the extra protection against bradycardia upon addition of INI-4001 is not statistically significant compared to F1-CRM + alum 24 µg. In addition, the TLR4 adjuvant, INI-2002, did not reduce bradycardia in comparison to F_1_-CRM or F_1_-CRM + alum, consistent with the lower F_1_ specific antibody levels measured in the serum of mice injected with F_1_-CRM + alum + INI-2002 compared to F_1_-CRM + alum + INI-4001. Finally, 30 min post-fentanyl challenge, blood and brain were collected for pharmacokinetic analysis to determine the concentration of fentanyl in the blood and brain. Mice injected with F_1_-CRM + alum + INI-4001 or with the triple adjuvant combination had a highly significant increase in serum fentanyl concentrations (Fig. 7c), with an associated significant decrease in brain fentanyl concentrations (Fig. 7d) when compared to F_1_-CRM only. Notably, brain fentanyl concentrations were below the level of detection in mice that were vaccinated with F_1_-CRM + alum + INI-4001. The addition of INI-2002 to alum + INI-4001 did not further increase serum fentanyl, suggesting that the protective effect arose from the use of alum + INI-4001 as a combination adjuvant with F_1_-CRM (Fig. 7c) as hypothesized based on the F_1_-specific antibody concentrations and avidity. As brain fentanyl concentration was undetectable in mice vaccinated with F_1_-CRM + alum + INI-4001, we cannot comment as to whether the addition of INI-2002 had any effect on brain fentanyl concentration. Overall, these data demonstrate that adjuvanting F_1_-CRM with alum + INI-4001 (TLR7/8 adjuvant) significantly increased the efficacy of F_1_-CRM as a fentanyl vaccine as evidenced mainly by undetectable brain fentanyl concentrations in mice vaccinated with F_1_-CRM + alum + INI-4001 while the addition of INI-2002 (TLR4 adjuvant) was not required to confer protective effects.

## Discussion

Here, we demonstrated that the use of a synthetic TLR7/8 adjuvant, INI-4001, in combination with alum significantly and preferentially increased F_1_-specific IgG2a antibody titers and significantly increased average polyclonal antibody avidity for the fentanyl hapten compared to F_1_-CRM and F_1_-CRM plus alum. When challenged with fentanyl, mice vaccinated with F_1_-CRM + INI-4001 + alum demonstrated a trend toward, but not statistically significant, increased protection against fentanyl-induced bradycardia compared to F_1_-CRM and F_1_-CRM plus alum, resulted in significantly increased serum fentanyl concentrations compared to F_1_-CRM and F_1_-CRM plus alum, and significantly decreased brain fentanyl concentrations compared to F_1_-CRM.

The addition of INI-2002, a synthetic TLR4 agonist, did not increase antibody responses or provide further protection from the fentanyl challenge. This may have been due to the finding that F_1_, CRM, and F_1_-CRM significantly reduced the ability of INI-2002 to activate TLR4. We were not able to find any reports in the literature regarding CRM-mediated inhibition of TLR4 and this phenomenon warrants further investigation. We measured endotoxin levels in each of the components (F_1_, CRM, and F_1_-CRM) and were not able to detect any. Previous data from vaccines targeting other opioids reported mixed responses when TLR4 agonists were used as adjuvants^20,25^. Others have also reported that opioids, such as morphine, act as TLR4 agonists and that TLR4 signaling contributes to the rewarding effects of morphine and other opioids^34,35^. Conversely, the µ opioid receptor (MOR) antagonists (−)-naloxone and (−)-naltrexone inhibit TLR4 activation by opioids^38,39^ while the non-MOR antagonists (+)-naloxone and (+)-naltrexone are also TLR4 antagonists that reduce the analgesic effects of morphine and reduce opioid withdrawal symptoms^35,40,41^. Both fentanyl and morphine have also been reported to inhibit TLR4 activation in certain contexts^36,37^. Given the ability of opioids and opioid haptens to bind TLR4, both as agonists and antagonists, TLR4-based adjuvants should be evaluated carefully when used in an opioid vaccine. Further, it remains an open question whether or not current opioid users would respond well to any TLR4 adjuvanted vaccine given their repeated exposure to compounds linked to TLR4 binding.

Previous publications on vaccines for opioids and OUD typically use an antigen dose (that is, hapten-protein conjugate) of 50–100 µg per mouse or rat and previous studies that investigated fentanyl vaccines specifically used a minimum of 25 ug fentanyl hapten-carrier conjugate per mouse or rat in combination with alum^7–10,12,13,21^. While 60 µg F_1_-CRM plus alum was found to almost completely prevent fentanyl from reaching the brain after three vaccinations^9^, here we found that while 5 µg F_1_-CRM was sufficient to protect mice against fentanyl-induced bradycardia and antinociception, the addition of alum+INI-4001 to 5 µg F_1_-CRM was necessary to further reduce brain fentanyl levels to undetectable levels after three vaccinations. This indicates a significant antigen dose-sparing effect and improved efficacy using alum+INI-4001 as a combination adjuvant for a fentanyl vaccine, which could have profound impacts on efficacy, durability, vaccine supply, and distribution.

While murine TLR7 and TLR8 do not perfectly recapitulate those of humans in terms of cell type expression and compound structure recognition, it is nonetheless generally true that murine TLR7 recognizes and responds to molecules that activate both human TLR7 and TLR8^27,42^. Additionally, the stimulation of murine TLR7 results in the production of pro-inflammatory cytokines along with type I IFNs, similar to that seen from stimulation of both human TLR7 and TLR8^42^. The human cytokine profile elicited by stimulation with INI-4001 gives us further confidence that INI-4001 will behave as a Th1-polarizing adjuvant in humans as well as mice based on the expression of type I IFNs^43,44^. Additionally, the expression of both IL-6 and type I IFNs has been shown to support T_FH_ differentiation^44^. Given the expression of IL-6 and type I IFNs by INI-4001 in hPBMCs, it is also possible that using INI-4001 as a human adjuvant will induce differentiation of T_FH_ cells, which supports high-affinity antibody production. 3M-052, a similar lipidated TLR7/8 agonist, generated strongly Th1-polarized vaccine responses in mice^45,46^ and nonhuman primates^47,48^. Additionally, 3M-052 was found to induce robust T_FH_ differentiation in rhesus macaques when combined with an HIV antigen^48^. Currently, 3M-052, as an adjuvant for an HIV vaccine, is in phase I clinical trials (NCT04177355). Although no human data have been reported yet, the similar Th1-polarized response and T_FH_ differentiation found in NHPs and mice after adjuvanting with a similar TLR7/8 agonist supports our hypothesis that INI-4001 will similarly polarize a Th1 immune response and support T_FH_ differentiation in humans. More recently, a SARS-CoV-2 vaccine that contains a TLR7/8 agonist, Covaxin, was approved for emergency use by the WHO for the prevention of COVID-19 with an overall estimated efficacy in preventing COVID-19 of 77.8%^49^, demonstrating the safety and efficacy of TLR7/8 agonists as vaccine adjuvants in a human population. Thus, we expect that INI-4001 will lead to the production of high affinity, high titer fentanyl-specific antibodies needed to prevent fentanyl from crossing the blood–brain barrier in people, making it a potent adjuvant in anti-fentanyl vaccines in the OUD population to treat OUD and prevent overdose deaths.

## Methods

### Hapten synthesis and conjugate vaccines

The fentanyl-based hapten (F_1_) containing a tetraglycine linker was synthesized as published in ref. ^8^ and conjugated to GMP grade diphtheria cross-reactive material (CRM; Pfenex, San Diego, CA) using carbodiimide chemistry^8,50,51^. Briefly, piperidone hydrochloride was alkylated with 2-(N-Boc-aminoethyl) bromide using potassium carbonate in acetonitrile to afford the N-substituted amino ethylpiperidine intermediate. Reductive amination of the ketone with aniline using sodium cyanoborohydride in the presence of an equimolar amount of acetic acid provided the 4-aminophenyl-piperidine precursor. The 4-aminophenyl piperidine precursor was acylated using propionyl chloride in the presence of Hunig’s base [*N*,*N*-diisopropylethylamine {DIPEA)]. Acid-mediated N-Boc terminal group deprotection followed by acylation with glutaric anhydride in the presence of pyridine yielded the carboxylic acid precursor. The linker (Gly)_4_-OtBu was then attached using 2- (1H-benzotriazol-1-yl)-1,1,3,3-tetramethyluronium hexafluorophosphate (HBTU) and DIPEA as coupling agents. Finally, the tert-butyl ester was hydrolyzed using 20% trifluoracetic acid in dichloromethane to provide hapten. The F_1_ hapten was then conjugated to either GMP grade diphtheria cross-reactive material (Pfenex, San Diego, CA or Fina Biosolutions, Rockville MD) or bovine serum albumin (BSA) using carbodiimide chemistry. haptens were first dissolved at a concentration of 5.2 nM in 0.1 M MES buffer pH 5.0 in the presence of 10% DMSO (w/v), and then haptens were activated by carbodiimide (EDAC, Sigma-Aldrich, St. Louis, MO) at a final concentration of 208 nM. The conjugation reaction was incubated at room temperature for 10 min before the carrier protein was added at a final concentration of 2.8 mg/ml. Reactions were then stirred for 3 h at room temperature. The resulting conjugates were purified by ultrafiltration using Amicon filters to replace MES buffer with phosphate-buffered saline (PBS) 0.1 M pH 7.2. For CRM conjugates, 250 mM sucrose was added in both conjugation and storage buffers for stability. For use as a coating antigen for ELISA, F_1_ hapten was conjugated to bovine serum albumin (BSA). Briefly, 5 mM F_1_ hapten was activated with 104 nM EDAC in 0.1 M MES buffer pH 5 with 250 mM sucrose and 10% DMSO. The reaction mixture was stirred for 10 min at room temperature. CRM was then added to a final concentration of 2.8 mg/mL and stirred for 3 h at room temperature. Using an Amicon filter unit (MilliporeSigma. Merck, Burlington, MA) with a 50 kDa molecular weight cutoff, MES buffer was exchanged for PBS buffer pH 7.2 with 250 mM sucrose. The conjugates were resuspended to a final concentration of 2.5 mg/mL. The haptenation ratio of BSA and CRM conjugates was measured by MALDI-TOF analysis (AB SCIEX 5800, Foster City, CA). The unconjugated carrier protein or the conjugate vaccines were adsorbed on aluminum adjuvant (alum; Alhydrogel® aluminum hydroxide (InvivoGen)) with or without various TLR agonists, as described in each experimental section.

### Drugs

Fentanyl citrate was obtained from Boynton Pharmacy (Minneapolis, MN).

### Synthesis of INI-2002

2-[(R)-3-decanoyloxytetradecanoylamino]ethyl 2,3-di-[(R)-3-decanoyloxytetradecanoylamino]-2,3-dideoxy-4-*O*-sulfoxy-β-d-allopyranoside was synthesized as follows:^52^

1. A solution of 1,3,4,6-tetra-*O*-acetyl-2-amino-2-deoxy-β-d-glucopyranose hydrochloride (76.47 g, 0.23 mol) in methylene chloride (350 mL) and H_2_O (350 mL) was treated with sodium bicarbonate (149.94 g, 1.79 mol) added in portions slowly. Benzyl chloroformate (79.17 g, 0.46 mol) was added in portions to control gas evolution and the reaction was stirred vigorously for 2.5 h. The layers were separated and the aqueous layer was extracted with methylene chloride (100 mL). The combined organic layers were washed with saturated aqueous sodium chloride, dried over anhydrous sodium sulfate, filtered and concentrated to ~100 mL. Methyl-*t*-butyl ether (200 mL) was added and the resulting mixture was stirred and cooled to 0 °C and the precipitate was collected by filtration, washed with cold methyl-*t*-butyl ether and dried in a vacuum oven to give 88.89 g (81 %) of 1,3,4,6-tetra-*O*-acetyl-2-(benzyloxycarbonylamino) 2-deoxy-β-d-glucopyranoside.
2. A solution of 1,3,4,6-tetra-*O*-acetyl-2-(benzyloxycarbonylamino) 2-deoxy-β-d-glucopyranoside (10 g, 20.8 mmol) and benzyl *N*-(2-hydroxyethyl)carbamate (4.48 g, 22.9 mmol) in anhydrous methylene chloride (80 mL), cooled to −15 °C, was treated dropwise with trimethylsilyl triflate (0.37 mL, 2.08 mmol). The reaction mixture was allowed to warm to room temperature over 5.5 h. The reaction was quenched with saturated aqueous sodium bicarbonate (40 mL) and the layers were separated. The aqueous layer was extracted with methylene chloride (2 × 20 mL) and the combined organic layers were dried over anhydrous sodium sulfate, filtered and concentrated in vacuo. The crude product obtained was crystallized from methylene chloride/heptane to give 10.4 g (81%) of 2-(benzyloxycarbonylamino)ethyl 3,4,6-tri-*O*-acetyl-2-benzyloxycarbonylamino-2-deoxy-β-d-glucopyranoside as a white solid.
3. A solution of 2-(benzyloxycarbonylamino)ethyl 3,4,6-tri-*O*-acetyl-2-benzyloxycarbonylamino-2-deoxy-β-d-glucopyranoside (10 g, 16.3 mmol) in methanol (160 mL) was treated with ammonium hydroxide (20 equivalents) for 2 h at room temperature. The reaction mixture was concentrated and dried under high vacuum overnight to give 8 g (100%) of 2-(benzyloxycarbonylamino)ethyl 2-benzyloxycarbonylamino-2-deoxy-β-d-glucopyranoside as a white solid, which was used without further purification.
4. A solution of 2-(benzyloxycarbonylamino)ethyl 2-benzyloxycarbonylamino-2-deoxy-β-d-glucopyranoside (8 g, 16.3 mmol) in acetonitrile (180 mL) was treated with benzaldehyde dimethyl acetal (4.9 mL, 32.6 mmol) and camphorsulfonic acid (1.9 g, 8.2 mmol). The reaction was stirred for 3 h, neutralized with saturated aqueous sodium bicarbonate, filtered and concentrated in vacuo. The crude product was crystallized from ethyl acetate/heptane to give 7.1 g (75%) of 2-(benzyloxycarbonylamino)ethyl 4,6-*O*-benzylidene-2-deoxy-2-benzyloxycarbonylamino-2-deoxy-β-d-glucopyranoside as a white solid.
5. A solution of 2-(benzyloxycarbonylamino)ethyl 4,6-*O*-benzylidene-2-deoxy-2-benzyloxycarbonylamino-2-deoxy-β-d-glucopyranoside (1.5 g, 2.59 mmol) in anhydrous tetrahydrofuran (40 mL) was treated with triethylamine (0.54 mL, 3.89 mmol) and triphenylphosphine (1.09 g, 4.14 mmol). The reaction mixture was cooled to 0 °C and diisopropyl azodicarboxylate (0.82 mL, 4.14 mmol) was added. After 45 min at 0 °C, diphenylphosphoryl azide (0.89 mL, 4.14 mmol) was added. The reaction was allowed to gradually warm up to room temperature and stirring continued for 18 h. The reaction mixture was concentrated in vacuo and the residue chromatographed on silica gel (gradient elution, 20→70 % ethyl acetate/heptane) affording 1.16 g (74 %) of 2-(benzyloxycarbonylamino)ethyl 3-azido-4,6-*O*-benzylidene-2-benzyloxycarbonylamino-2,3-dideoxy-β-d-allopyranoside as a white solid.
6. A solution of 2-(benzyloxycarbonylamino)ethyl 3-azido-4,6-*O*-benzylidene-2-benzyloxycarbonylamino-2,3-dideoxy-β-d-allopyranoside (2.95 g, 4.89 mmol) in anhydrous tetrahydrofuran (100 mL) was treated with a solution of 0.1 N sodium hydroxide (9.8 mL, 0.98 mmol) and a solution of 1.0 M of trimethylphosphine in tetrahydrofuran (7.8 mL, 7.82 mmol). The reaction stirred at room temperature for 18 h. The reaction mixture was concentrated in vacuo. The residue was chromatographed on silica gel (gradient elution, 30→100 % ethyl acetate/heptane then 0→10% methanol/chloroform) affording 2.37 g (84 %) of 2-(benzyloxycarbonylamino)ethyl 3-amino-4,6-*O*-benzylidene-2-benzyloxycarbonylamino-2,3-dideoxy-β-d-allopyranoside as a white solid.
7. A solution of 2-(benzyloxycarbonylamino)ethyl 3-amino-4,6-*O*-benzylidene-2-benzyloxycarbonylamino-2,3-dideoxy-β-d-allopyranoside (0.5 g, 0.87 mmol) in anhydrous methylene chloride (10 mL) was acylated with (*R*)-3-decanoyloxytetradecanoic acid (414 mg, 1.04 mmol) and 1-(3-dimethylaminopropyl)-3-ethylcarbodiimide methiodide (310 mg, 1.04 mmol) at room temperature for 2 h. The reaction mixture was quenched with saturated aqueous sodium bicarbonate (5 mL) and the layers separated. The aqueous layer was extracted with chloroform (2 × 5 mL) and the combined organic layers were washed with water (5 mL), dried over anhydrous sodium sulfate and concentrated in vacuo. Chromatography on silica gel (gradient elution, 10→ 60% ethyl acetate/heptane) afforded 748 mg (90 %) of 2-(benzyloxycarbonylamino)ethyl 4,6-*O*-benzylidene-2-benzyloxycarbonylamino-3-[(*R*)-3-decanoyloxytetradecanoylamino]-2,3-dideoxy-β-d-allopyranoside as a colorless oil.
8. A solution of 2-(benzyloxycarbonylamino)ethyl 4,6-*O*-benzylidene-2-benzyloxycarbonylamino-3-[(*R*)-3-decanoyloxytetradecanoylamino]-2,3-dideoxy-β-d-allopyranoside (745 mg, 0.78 mmol) in anhydrous tetrahydrofuran (20 mL) was hydrogenated with 10% palladium on carbon (220 mg) using a Parr hydrogenator at room temperature and 50 psig for 24 h. The reaction mixture was filtered through Celite and the filtrate concentrated in vacuo. The resulting oil dissolved in methylene chloride (10 mL) was acylated with (*R*)-3-decanoyloxytetradecanoic acid (680 mg, 1.71 mmol) and 1-(3-dimethylaminopropyl)-3-ethylcarbodiimide methiodide (510 mg, 1.71 mmol) at room temperature for 2 h. The reaction mixture was quenched with saturated aqueous sodium bicarbonate (10 mL) and the layers separated. The aqueous layer was extracted with methylene chloride (2 × 10 mL) and the combined organic layers washed with water (10 mL), dried over anhydrous sodium sulfate and concentrated in vacuo. Chromatography on silica gel (gradient elution, 20→80% ethyl acetate/heptane) afforded 732 mg (65%) of 2-[(*R*)-3-decanoyloxytetradecanoylamino]ethyl 4,6-*O*-benzylidene-2,3-di-[(*R*)-3-decanoyloxytetradecanoylamino]-2,3-dideoxy-β-d-allopyranoside as a glassy solid.
9. A solution of 2-[(*R*)-3-decanoyloxytetradecanoylamino]ethyl 4,6-*O*-benzylidene-2,3-di-[(*R*)-3-decanoyloxytetradecanoylamino]-2,3-dideoxy-β-d-allopyranoside (400 mg, 0.282 mmol) in anhydrous methylene chloride (20 mL) cooled to 0 °C was treated with sodium cyanoborohydride (42 mg, 0.655 mmol) followed by the addition of trifluoroacetic acid (0.06 mL, 0.786 mmol). The reaction mixture gradually warmed up to room temperature and continued to stir for 3 h. The reaction was quenched with methanol (2 mL), concentrated in vacuo, then reconstituted in methylene chloride, and washed with a saturated solution of sodium bicarbonate. The layers separated and the aqueous layer was extracted with methylene chloride (2 × 10 mL) and the combined organic layers dried over anhydrous sodium sulfate and concentrated in vacuo. Chromatography on silica gel (gradient elution, 10→ 95% ethyl acetate/heptane) afforded 380 mg (93%) of 2-[(*R*)-3-decanoyloxytetradecanoylamino]ethyl 6-*O*-benzyl-2,3-di-[(*R*)-3-decanoyloxytetradecanoylamino]-2,3-dideoxy-β-d-allopyranoside as a colorless oil.
10. A solution of 2-[(*R*)-3-decanoyloxytetradecanoylamino]ethyl 6-*O*-benzyl-2,3-di-[(*R*)-3-decanoyloxytetradecanoylamino]-2,3-dideoxy-β-d-allopyranoside (105 mg, 0.072 mmol) in anhydrous dimethylformamide (5 mL) was treated with sulfur trioxide triethylamine complex (78 mg, 0.43 mmol). The reaction was heated to 50 °C for 5 h. An additional amount of sulfur trioxide triethylamine complex (100 mg, 0.55 mmol) was added and the reaction was stirred at 50 °C for 18 h. The reaction mixture was concentrated in vacuo. Chromatography on C_18_ column (gradient elution, 5→ 20 % methylene chloride + 1% triethylamine/methanol) afforded 90 mg (82%) of 2-[(*R*)-3-decanoyloxytetradecanoylamino]ethyl 6-*O*-benzyl-2,3-di-[(*R*)-3-decanoyloxytetradecanoylamino]-2,3-dideoxy-4-*O*-sulfoxy-β-d-allopyranoside triethylammonium salt as a white salt.
11. A solution of 2-[(*R*)-3-decanoyloxytetradecanoylamino]ethyl 6-*O*-benzyl-2,3-di-[(*R*)-3-decanoyloxytetradecanoylamino]-2,3-dideoxy-4-*O*-sulfoxy-β-d-allopyranoside triethylammonium salt (70 mg, 0.045 mmol) in a mixture of 2:1 anhydrous tetrahydrofuran: methanol (5 mL) was hydrogenated in the presence of 20% palladium hydroxide on carbon (30 mg) and triethylamine (0.034 mL, 0.00024 mmol) using a Parr hydrogenator at room temperature and 50 psig pressure for 18 h. The reaction mixture was filtered through Celite and the filtrate was concentrated under vacuum. Chromatography on C_18_ silica column (gradient elution, 5→20 % methylene chloride + 1% triethylamine/methanol), the purified material was dissolved in cold 2:1 chloroform-methanol (8 mL) and washed with cold 0.1 N aqueous hydrochloride (1.6 mL). The lower organic layer was dried over anhydrous sodium sulfate and concentrated in vacuo. The residue was salted with (1–2 equiv.) triethylamine to give 28 mg (43%) of 2-[(*R*)-3-decanoyloxytetradecanoylamino]ethyl 2,3-di-[(*R*)-3-decanoyloxytetradecanoylamino]-2,3-dideoxy-4-*O*-sulfoxy-β-d-allopyranoside triethylammonium salt as a glassy solid: ^1^H NMR (CDCl_3_/CD_3_OD): δD): (CDClD-allo*J* = 5.5 Hz, 1 H), 7.55 (d, *J* = 8.0 Hz, 1 H), 7.22 (d, *J* = 9.0 Hz, 1 H), 5.27 – 5.23 (m, 3 H), 4.65 (br s, 1 H), 4.59 – 4.55 (m, 2 H), 4.26 – 4.21 (m, 1 H), 4.19– 4.15 (m, 1 H), 3.85 – 3.79 (m, 2 H), 3.73 – 3.70 (m, 1 H), 3.51 – 3.43 (m, 2 H), 3.18 (q, *J* = 7.5 Hz, 7 H, CH_2_ of triethylamine ( ~ 1.2 equiv.)), 2.62 – 2.19 (m, 12), 1.64 – 1.52 (m, 12 H), 1.37 – 1.26 (m, 100 H, including 10, CH_3_ of triethylamine), 0.88 (t, *J* = 7.0 Hz, 18 H); HRMS (ESI-TOF) m/z: Calcd for C_80_H_151_N_3_O_16_S [M-H]^-^ 1441.0737, found 1441.0714.

### Synthesis of INI-4001

INI-4001 was synthesized by phospholipidation of 6-amino-2-butoxy-9-[(1-hydroxyethyl-4-piperidinyl)-methyl]-7, 9-dihydro-8H-purin-8-one^53,54^. All dry reagents were dried from anhydrous pyridine or toluene and left under a high vacuum for 18 h. All glassware was heat dried and purged with dry, inert gas. The first two steps were done under argon. To a solution of PEG3 glycerol (1.16 g, 1.65 mmol) and 2-cyanoethyltetraisopropyl-phosphordiamidite (523 µL, 1.65 mmol) in 20 mL anhydrous methylene chloride was added 1H-tetrazole (115 mg, 1.65 mmol) in four portions over 20 min. After stirring at room temperature for 1 h, the reaction was cooled in an ice bath. To the cooled solution was added UM-3002 (500 mg, 1.20 mmol) and imidazolium triflate (525 mg, 2.41 mmol). After 10 min, the reaction was removed from the ice bath and stirred at room temperature for one hour. Tert-butyl peroxide (438 µL, 5.5 M in nonane) was added and the reaction was stirred at room temperature for 30 min. The reaction was quenched with saturated sodium thiosulfate and the organic layer was dried over sodium sulfate and condensed. The crude material was dissolved in 20 mL methylene chloride and DBU (1.80 mL, 12.1 mmol) was added. After 15 min, the solution was quenched with 0.1 N HCl and the organic layer dried over sodium sulfate. Purification on silica ([acetonitrile/methanol 1:1]/chloroform) afforded INI-4001 (680 mg, 50%) as an off-white solid. 1H NMR (400 Hz) δ 5.19-5.21 (m, 1H); 5.06-5.10 (m, 1H); 4.34 (dd, J = 11.8 Hz, 1H); 4.12-4.19 (m, 3H); 4.04 (dt, 6.6 Hz, 2H); 3.58-3.77 (m, 14H); 3.26 (bs, 2H); 2.77(bs, 1H); 2.25-2.34 (m, 5H); 1.74-1.95 (m, 4H); 1.72-1.74 (m, 1H); 1.52-1.61 (m, 5H); 1.37-1.47 (m, 3H); 1.25-1.31 (m, 50H); 0.93 (t, 7.2 Hz, 3H); 0.88 (t, 6.8 Hz, 6H). Anal. Calcd for C59H109N6O13P: C (62.08), H (9.63), N (7.36). Found: C (61.79), H (9.71), N (7.24).

### HEK293 assays

Human TLR4, TLR7, or TLR8 expressing HEK cells were obtained from Invivogen (San Diego, CA) or Novus (human TLR7 only). Cells were cultured according to the manufacturer’s instructions in DMEM with 10% FBS and selection antibiotics. HEK cells were plated at a density of 1 × 10^5^ cells/well in a flat bottom 96-well plate and incubated for 48 h at 37° C with indicated concentrations of INI-2002, INI-4001, F_1_, CRM, F_1_-CRM, or indicated combination. Cell supernatants were harvested and analyzed for NFκB activity via SEAP production following the manufacturer’s instructions using the QuantiBlue kit (Invivogen). SEAP activity was assessed by reading the optical density (OD) at 620–655 nm with a microplate reader. Data are expressed as the fold change in OD over vehicle-treated cells.

### PBMC isolation

Human blood was obtained from healthy adult donors through a University of Montana Institutional Review Board (IRB)-approved protocol. Informed consent was obtained from all donors. Peripheral blood mononuclear cells (hPBMCs) were separated from whole blood via density gradient separation using Histopaque 1077 (Sigma). For PBMC-based assays, cells were resuspended at the desired cell concentration in complete media (RPMI1640 + 5% autologous donor plasma). Cells were stimulated with the indicated compound concentrations and incubated for 24 h, at which point supernatants were harvested and analyzed for secreted cytokines by ELISA or MSD.

### Secreted cytokine assays

For experiments using human peripheral blood mononuclear cells (hPBMCs), supernatants were harvested after 24 h of stimulation with indicated concentration of compound or vehicle. Harvested supernatants were analyzed for TNFα, IFNβ, MIP-1α, IL-6, MCP-1, IP-10/CXCL10, and IL-1β using a custom U-PLEX MesoScale Discovery (MSD) assay according to the manufacturer’s instructions. IFNα was analyzed via VeriKine-HS Interferon Alpha All Subtype ELISA Kit (PBL Assay Science) according to the manufacturer’s instructions. For experiments using disaggregated mouse splenocytes, splenocytes were incubated with 1 µg/mL F_1_-CRM for 72 h. Supernatants were then harvested and analyzed for mouse IFNγ and IL-5 using a custom U-PLEX MSD assay according to the manufacturer’s instructions.

### Alum adsorption

F_1_-CRM, INI-4001, and alum were mixed using mouse in vivo study doses (5 µg F_1_-CRM, 10 µg INI-4001, 22.5 µg Alhydrogel® aluminum hydroxide (InvivoGen) in 2% glycerin in water for irrigation (WFI)). At 5, 15, 30, and 60 min, a sample was centrifuged at 14000 rcf for 5 min, and the amount of free F_1_-CRM and INI-4001 in the supernatant was measured. To measure free F_1_-CRM and INI-4001, a calibration curve was created by measuring the OD at 280 nm of samples with increasing concentrations of F_1_-CRM or INI-4001 in 2% glycerin in WFI. The percent of free F_1_-CRM or INI-4001 in supernatants was then determined by comparing the measured OD at 280 nm of the sample to the calibration curve.

### Animals

Six- to eight-week-old female Balb/c mice were obtained from Jackson Laboratory (Bar Harbor, ME). Animals were group-housed under a standard 12/12 h (University of Montana) or 14/10 h (University of Minnesota) light/dark cycle and were given food and water ad libitum. All testing occurred during the light phase.

### In vivo mouse studies

Animal studies were carried out in an OLAW and AAALAC-accredited vivarium in accordance with the University of Montana and the University of Minnesota’s IACUC guidelines for the care and use of laboratory animals. All studies were approved by the University of Montana IACUC or the University of Minnesota IACUC. Groups of five to eight female BALB/c mice were vaccinated intramuscularly on days 0 and 14 with 5 µg F_1_-CRM (based on a mass of CRM) with indicated amounts of Alhydrogel® aluminum hydroxide (InvivoGen), INI-2002, INI-4001 or various combinations as noted in 50 µL total volume per injection (compounds and antigen were diluted as needed in 2% glycerol in water). For mice that were not challenged with fentanyl, all animals were euthanized on day 28 and cardiac punctures were performed for F_1_-specific antibody analysis, and spleens were harvested for F_1_-CRM-specific T-cell analysis. In experiments where mice were challenged with fentanyl, only male mice were used. Mice were challenged subcutaneously (SC) on day 21 (7 days after the second injection), received a third injection on day 28, and challenged SC on day 35 (7 days after the third injection). Fentanyl challenge was performed with 0.05 mg/kg fentanyl. Post-challenge, reductions in bradycardia using a MouseOX PLus pulse oximeter (Starr Life Sciences, Oakmont, PA) and antinociception using a hotplate (Columbus Instruments, Columbus, OH) were measured^9,55^. A collar pulse oximeter was placed on the mouse using the manufacturer’s instructions at the indicated timepoints to measure bradycardia. Immediately after, the mouse was placed on a hotplate set to 54 °C and time to response was measured, defined as either a lift or flick of either hindpaw. Mice were removed from the hotplate if they did not respond within 60 s to prevent thermal injury. Thirty minutes after the final challenge, blood and brain were collected for pharmacokinetic analysis to determine fentanyl concentration using triple quadrupole LC/MS (Agilent)^9^. Brain tissue was homogenized using Agilent ceramic beads with a Beadblaster 24 homogenizer (Benchmark Scientific, Sayreville, NJ) and then centrifuged for 10 min at 8609×*g*. The resulting homogenate was transferred to a cryogenic tube and stored at −20 °C until extraction. Opioids from serum, brain, and standards were extracted at 4 °C. Cold LCMS grade Acetonitrile was added to all tubes to precipitate proteins and then centrifuged at 8609×*g* for 10 min. The supernatant was transferred to 96-well plates, evaporated using a Positive Pressure Manifold, Agilent (Santa Clara, CA), and then diluted with 2% phosphoric acid. Extraction was performed using Bond Elut PCX 96 round, 1 mL, 30 mg plate (Agilent, Santa Clara, CA). Prior to loading samples, cartridges were first washed with 500 μL methanol and 500 μL water. Cartridges were then washed with 2% formic acid followed by 50% methanol:50% acetonitrile. Cartridges were dried on a sample concentrator (Porvair, Norfolk, UK), eluted into a new 96-well plate using 5% ammonium hydroxide in 50% methanol: 50% acetonitrile, and dried on the sample concentrator. Samples were reconstituted in 200 μL LCMS grade water, 0.1% ammonium formate, and 0.01% LCMS grade formic acid (mobile phase A).

### F_1_-specific antibody concentrations

Opioid-specific IgG and IgG subclass titers were measured via ELISA^8,56^. Ninety-six-well ELISA plates (Costar 9018 EIA/RIA, Jackson ImmunoResearch Laboratories Inc., West Grove, PA) were coated with 5 ng/well of unconjugated BSA or F_1_ hapten conjugated to BSA in carbonate buffer at pH 9.6 overnight. The following day, plates were blocked with 1% gelatin. Mouse serum was added to wells and serially diluted. Plates were then incubated overnight with the following secondary antibodies: goat anti-mouse IgG-HRP (Jackson ImmunoResearch, West Grove, PA, catalog #115-035-003), goat anti-mouse IgG1-HRP or IgG2a-HRP (Alpha Diagnostic International, Inc., San Antonio, TX, catalog #40126 and 40127, respectively). All secondary antibodies were diluted at 1:30,000. Plates were developed using SIGMAFAST OPD substrate (Sigma-Aldrich, St. Louis, MO). In Figs. 3,4, F_1_ antibody concentrations were determined using an internal standard consisting of pooled serum from F_1_-CRM vaccinated mice where the F_1_ antibody concentration of undiluted serum was arbitrarily set to 1000 antibody units/mL. The same pooled serum was used as an internal standard for all ELISAs in Figs. 3, 4. In Supplementary Fig. 5, antibody titers were determined using a 1/dilution factor at the midpoint of the serum dilution curve.

### CRM-specific antibodies

MaxiSorp Nunc ELISA plates were coated with CRM-197 at 12.5 µg/mL in DPBS in 100 µL/well overnight at room temperature. Plates were washed (PBS with 0.05% Tween-20) and blocked with SuperBlock (ScyTek) for 1 h at 37 °C and then removed. Samples were serially diluted in EIA (PBS + 1% BSA, 0.1% Tween-20, 5% heat-inactivated FBS) down the plate and incubated at 37 C for 1 h. Plates were washed and 100 µL goat anti-mouse IgG Total-HRP (Southern Biotech, catalog #1036-05) antibody at 1:4000 was added to each well and incubated for 1 h at 37 °C. Plates were washed and 100 µl of room temperature SureBlue TMB Peroxidase (KPL) substrate was added to each well and incubated at room temperature for 1 h. Plates were read at 650 nm.

### PEG-specific antibodies

Serum from vaccinated mice was analyzed using a commercially available competitive ELISA kit that measures the concentration of PEGylated proteins following the manufacturer’s protocol (Abcam, Cambridge, UK). Serially diluted serum samples were mixed with HRP-conjugated PEG and added to a 96-well plate pre-coated with anti-PEG antibody. Standards were prepared as detailed in the manufacturer’s protocol. Samples and controls were incubated for 45 min with shaking and then washed to remove excess reagents. Plates were incubated with 100 µL TMB per well, stopped with assay stop solution, and read on a plate reader at 450 nm. PEG concentrations were calculated based on average absorbance by fitting a trend line to the standard curve generated OD values and analysis was done using statistical fitting software (XLfit, IDBS, Alameda, CA). A decrease in the measured PEG concentration indicates the presence of anti-PEG antibodies in mouse serum samples.

### Antibody avidity

Antibody avidity assays were performed on an Octet Red 96e instrument (Fortebio) at 30 °C with shaking at 1000 rpm in a solid black 96-well plate (Grenier). In vivo serum samples (analyte) and biotinylated antigen (ligand, F_1_-biotin), were diluted in 10X Kinetic Buffer (Fortebio). 10X Kinetic Buffer was used for ligand and analyte dilutions and all subsequent steps to avoid signal variations from step to step. Assays were performed by loading F_1_-biotin onto pre-hydrated streptavidin sensors at 0.1 µg/ml (loading step: 120 s) followed by a 180 s baseline. Sensors were then moved into the analyte to allow for the association for 120 s. All mouse serum samples were run at three different dilutions; serum antibody concentrations were normalized to equivalent concentrations across samples based on IgG anti-F1 antibody units and were diluted accordingly. The association of the antibody to the antigen was followed by a 600 s dissociation step. Dissociation rate constants (*K*_diss_) were calculated by processing raw data using ForteBio HT analysis software version 11.1.3.50. All data were inspected for quality of fit to the calculated curve (*R*^2^ > 0.95), the response between 0.25–3 (nm shift), and residual value <10% of the maximum response fitted to the curve.

### Reporting summary

Further information on research design is available in the Nature Research Reporting Summary linked to this article.

## Supplementary information

Supplemental material

REPORTING SUMMARY

## Data Availability

The datasets generated for this study are available on request to the corresponding author, Dr. Jay Evans (jay.evans@mso.umt.edu).
