# Supplementary material for: A lipidated TLR7/8 adjuvant enhances the efficacy of a vaccine against fentanyl in mice
Source: NPJ Vaccines. 2023 Jul 10;8:97. doi: 10.1038/s41541-023-00694-y (PMC10333387; doi:10.1038/s41541-023-00694-y)
Supplement: Supplementary file 1 — Supplemental material [file 41541_2023_694_MOESM1_ESM.pdf]

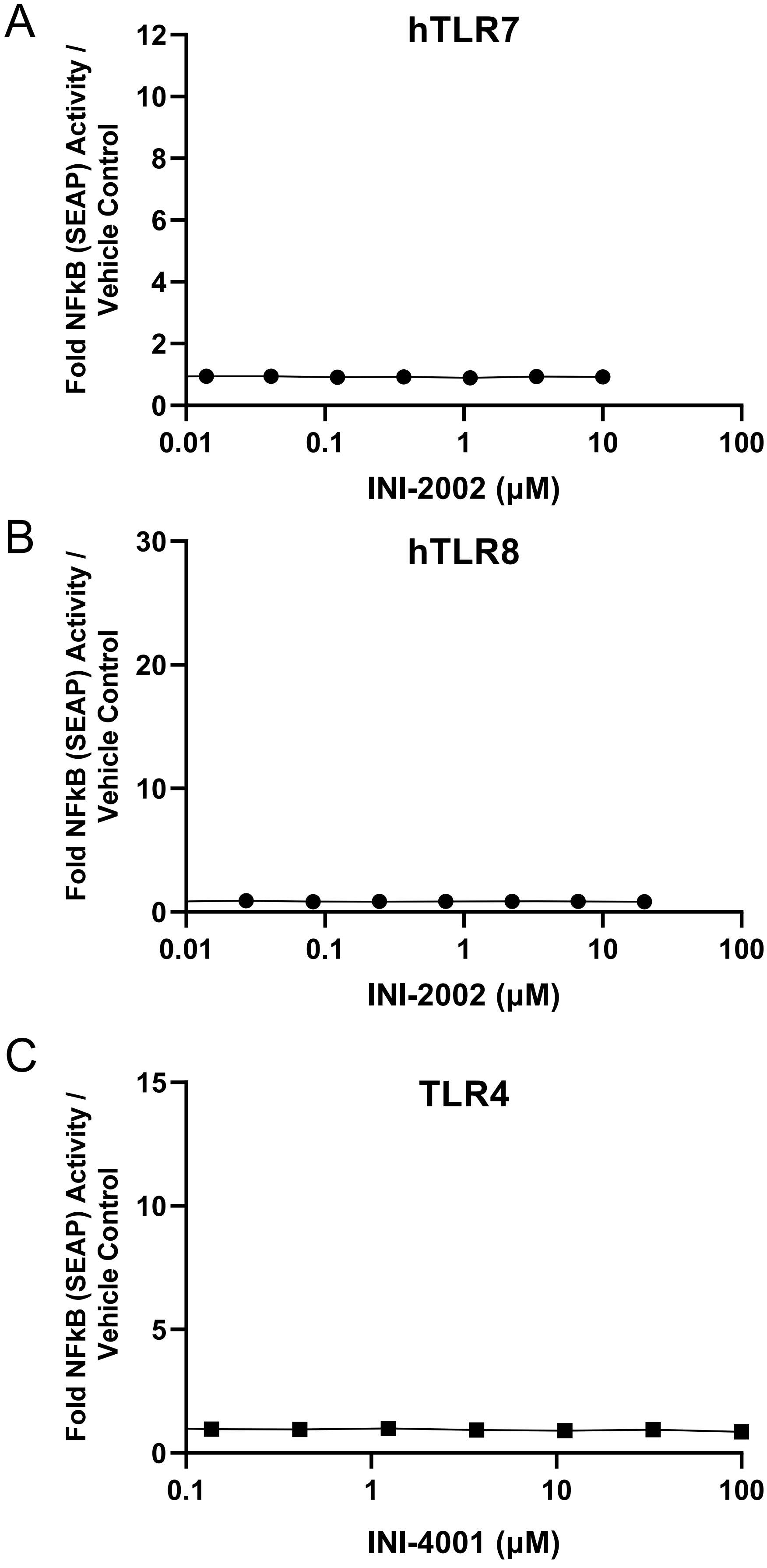

Supplementary Figure 1: Activation of NF-κB in HEK cells A) stimulation of HEK cells expressing TLR7 with INI-2002, B) stimulation of HEK cells expressing TLR8 with INI-2002, and C) stimulation of HEK cells expressing TLR4 with INI-4001. Data are shown as mean +/- SEM.

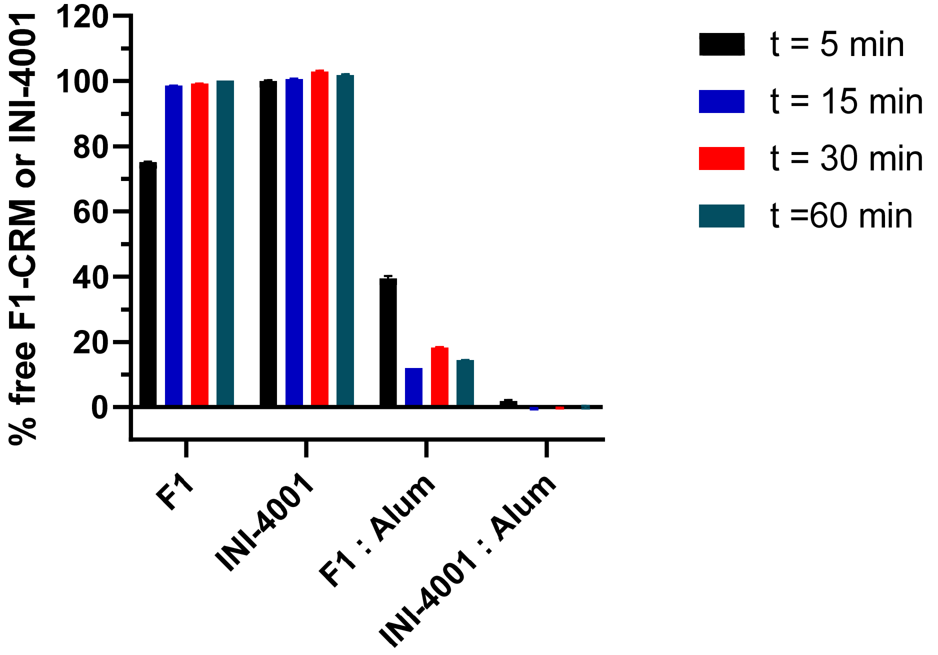

Supplementary Figure 2: F1-CRM and INI-4001 are fully adsorbed to alum after 5 minutes of incubation. F1-CRM, INI-4001 and alum were mixed using mouse in vivo study doses (5 µg F_1-CRM, 10 µg INI-4001, 22.5 µg Alhydrogel® aluminum hydroxide (InvivoGen) in 2% glycerin in water for irrigation (WFI)). At 5, 15,30 and 60 minutes, a sample was centrifuged at 14000 rcf for 5 minutes and the amount of free F_1-CRM and INI-4001 in the supernatant was measured. Data are shown as mean +/- SEM.

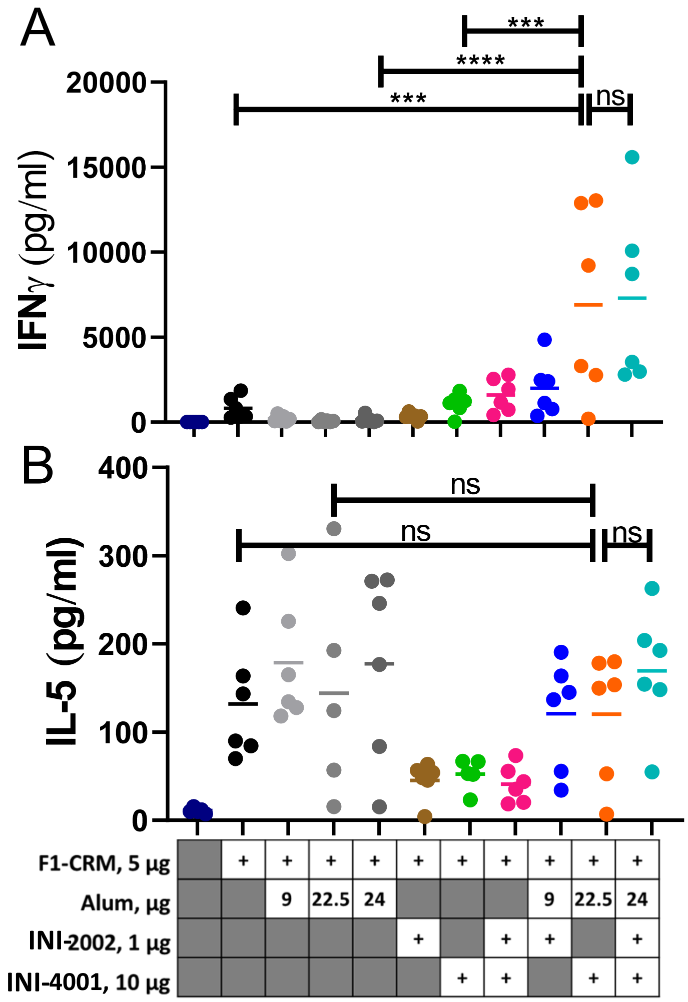

Supplementary Figure 3: CRM-specific T cell responses support F_1-specific antibody responses and class switching. Mice were vaccinated twice, IM, with 5 µg F_1-CRM plus 9, 22.5, or 24 µg alum, 1 µg INI-2002, 10 µg INI-4001, the combination of INI-2002 + INI-4001, INI-2002 + alum, INI-4001 + alum, or INI-2002 + INI-4001+ alum as indicated. 14 days after the second vaccination, spleens were harvested and disaggregated. Splenocytes were restimulated for 72 hours with 1 ug/mL CRM at which point supernatants were harvested and analyzed for IFNγ and IL-5 using a custom U-PLEX MSD assay. Statistical analysis conducted by one-way ANOVA with Fisher's LSD for multiple comparisons (GraphPad Prism) where * = p≤0.05, ** = p≤0.01, *** = p≤0.001, **** = p≤0.0001.

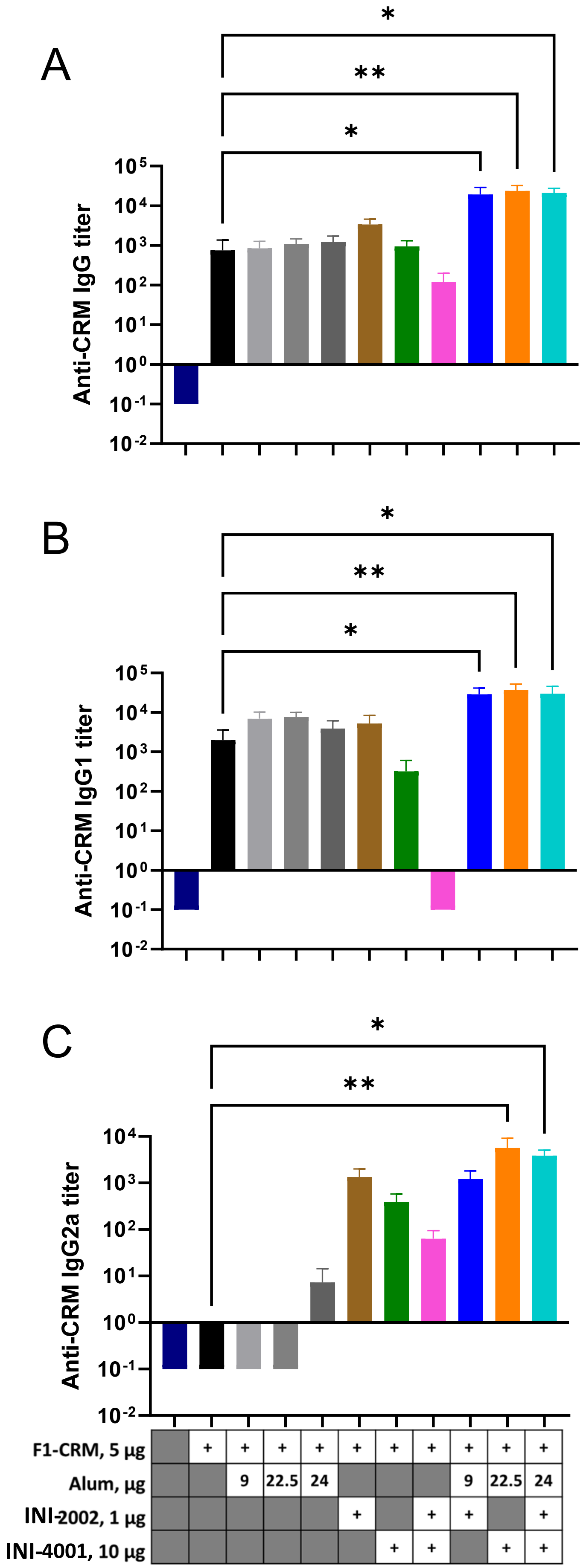

Supplementary Figure 4: Alum, INI-2002, and INI-4001 adjuvants affect CRM-specific antibody responses similarly to F1-specific antibody responses. Mice were vaccinated twice, IM, with 5 µg F1-CRM plus 9, 22.5, or 24 µg alum, 1 µg INI-2002, 10 µg INI-4001, the combination of INI-2002 + INI-4001, INI-2002 + alum, INI-4001 + alum, or INI-2002 + INI-4001 + alum as indicated. 14 days after the second vaccination, blood was collected and anti-F1 IgG (A), IgG1 (B), and IgG2a (C) antibody concentrations were measured by ELISA. Statistical analysis conducted by one-way ANOVA with Fisher's LSD for multiple comparisons (GraphPad Prism). * = p≤0.05, ** = p≤0.01, *** = p≤0.001, **** = p≤0.0001; color of asterisks indicates comparison group. Data are shown as mean +/- SEM.

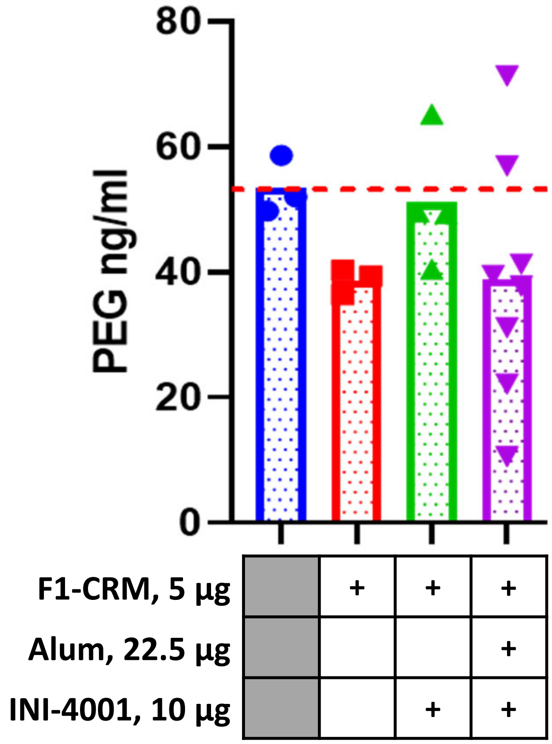

Supplementary Figure 5: PEG-specific antibodies do not increase after vaccination of F_1-CRM+INI-4001, a PEGylated compound. Mouse serum samples from Fig. 3 were used in a PEG competitive ELISA. Decreased detection of PEG in vaccinated mice compared to unvaccinated mice indicates the presence of PEG-specific antibodies. Individual data points are shown along with bars representing the mean.

Fig S4 octet raw data

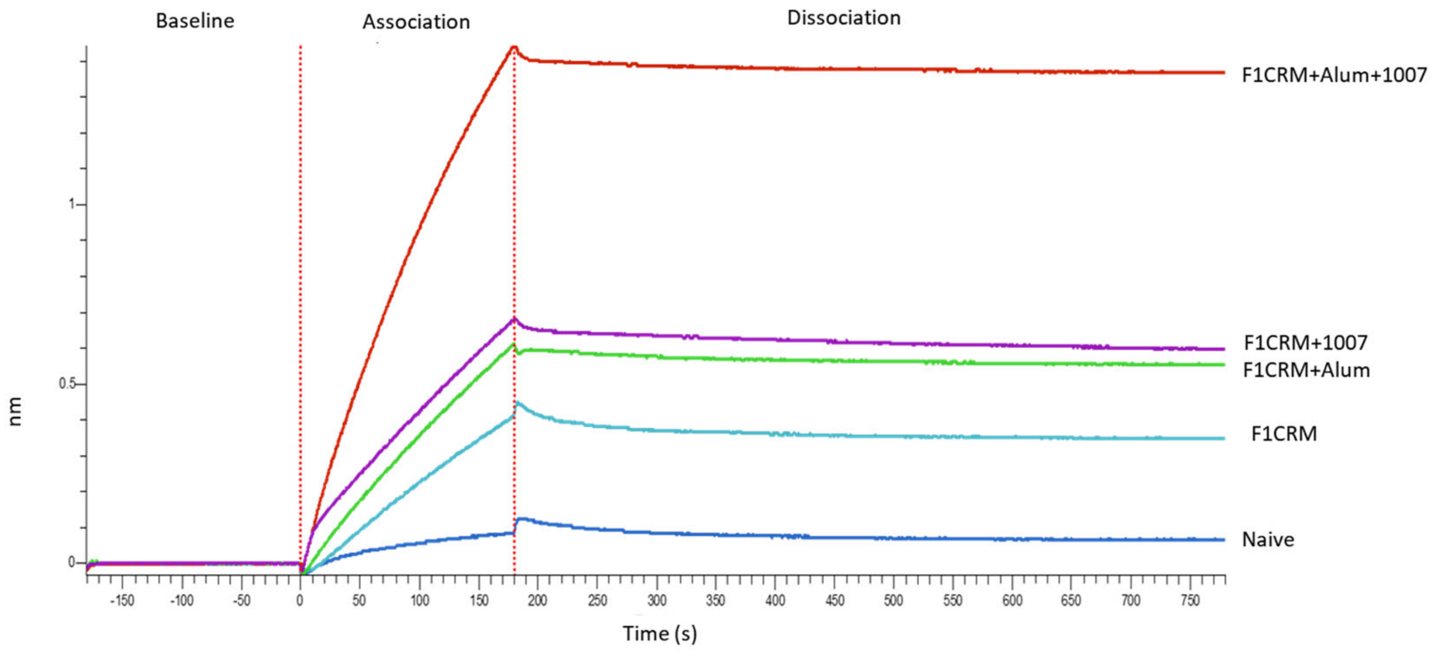

Supplementary Figure 6. Representative Octet sensorgram of polyclonal anti-fentanyl antibody responses. The sensorgram shows the interaction of polyclonal serum (analyte) and biotinylated F_1 antigen (ligand) loaded on the sensor tip during association and dissociation steps. Representative sensorgrams are shown for one sample from the indicated vaccination groups.

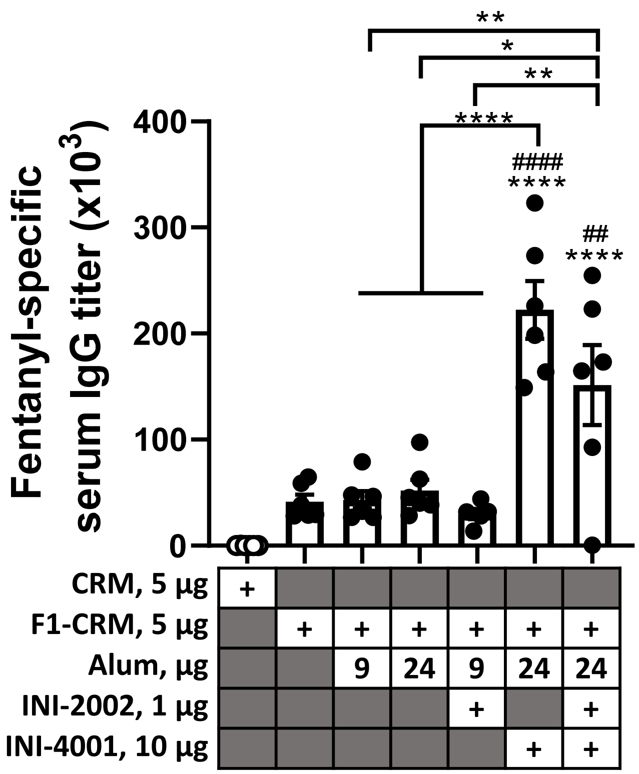

Supplementary Figure 7: Pre-fentanyl challenge antibody titers after 3 vaccinations follow the same trends as after 2 vaccinations. Mice were immunized, IM, on days 0, 14, and 28. On day 34, prior to fentanyl challenge, mice were bled and F_1-specific antibody titers were determined in serum using 1/dilution factor at the midpoint of the serum dilution curve. Statistical analysis conducted by one-way ANOVA with Tukey's multiple comparisons post hoc test. Symbols: * $p \leq 0.05$, ** or ## $p \leq 0.01$, *** $p \leq 0.001$, **** or #### $p \leq 0.0001$ compared to control (*) or F_1-CRM (#). Individual data points are shown along with bars representing the mean and error bars representing SEM.
