# Supplementary material for: A lipidated TLR7/8 adjuvant enhances the efficacy of a vaccine against fentanyl in mice
Source: NPJ Vaccines. 2023 Jul 10;8:97. doi: 10.1038/s41541-023-00694-y (PMC10333387; doi:10.1038/s41541-023-00694-y)
Supplement: Supplementary file 2 — REPORTING SUMMARY [file 41541_2023_694_MOESM2_ESM.pdf]

nature portfolio

Corresponding author(s): Jay T Evans

Last updated by author(s): Nov 21, 2022

Reporting Summary

Nature Portfolio wishes to improve the reproducibility of the work that we publish. This form provides structure for consistency and transparency in reporting. For further information on Nature Portfolio policies, see our Editorial Policies and the Editorial Policy Checklist.

Statistics

For all statistical analyses, confirm that the following items are present in the figure legend, table legend, main text, or Methods section.

n/a	Confirmed	
☐	☒	The exact sample size (n) for each experimental group/condition, given as a discrete number and unit of measurement
☐	☒	A statement on whether measurements were taken from distinct samples or whether the same sample was measured repeatedly
☐	☒	The statistical test(s) used AND whether they are one- or two-sided<br>*Only common tests should be described solely by name; describe more complex techniques in the Methods section.*
☒	☐	A description of all covariates tested
☐	☒	A description of any assumptions or corrections, such as tests of normality and adjustment for multiple comparisons
☐	☒	A full description of the statistical parameters including central tendency (e.g. means) or other basic estimates (e.g. regression coefficient) AND variation (e.g. standard deviation) or associated estimates of uncertainty (e.g. confidence intervals)
☐	☒	For null hypothesis testing, the test statistic (e.g. F, t, r) with confidence intervals, effect sizes, degrees of freedom and P value noted<br>*Give P values as exact values whenever suitable.*
☒	☐	For Bayesian analysis, information on the choice of priors and Markov chain Monte Carlo settings
☒	☐	For hierarchical and complex designs, identification of the appropriate level for tests and full reporting of outcomes
☒	☐	Estimates of effect sizes (e.g. Cohen's d, Pearson's r), indicating how they were calculated

Our web collection on statistics for biologists contains articles on many of the points above.

Software and code

Policy information about availability of computer code

Data collection	No software was used for data collection
Data analysis	Statistical analyses were performed using GraphPad Prism. XLfit was used for curve fitting of ELISA data.

For manuscripts utilizing custom algorithms or software that are central to the research but not yet described in published literature, software must be made available to editors and reviewers. We strongly encourage code deposition in a community repository (e.g. GitHub). See the Nature Portfolio guidelines for submitting code & software for further information.

Data

Policy information about availability of data

All manuscripts must include a data availability statement. This statement should provide the following information, where applicable:

- Accession codes, unique identifiers, or web links for publicly available datasets
- A description of any restrictions on data availability
- For clinical datasets or third party data, please ensure that the statement adheres to our policy

The datasets generated for this study are available on request to the corresponding author, Dr Jay Evans (jay.evans@mso.umt.edu).

Human research participants

Policy information about studies involving human research participants and Sex and Gender in Research.

Reporting on sex and gender	Human blood donors are reported by blood donor number only. While there are both males and females in the blood donor pool, the authors have no knowledge regarding the sex of a given blood donor.
Population characteristics	All blood donors are de-identified. As such, the authors have no knowledge regarding individual blood donors other than donor number.
Recruitment	Any healthy adult ages 18-65 is eligible to participate. Participants were recruited via word of mouth at the University of Montana. Thus, donors may skew toward the age and demographics of the University of Montana community.
Ethics oversight	The study protocol was approved by the University of Montana IRB.

Note that full information on the approval of the study protocol must also be provided in the manuscript.

Field-specific reporting

Please select the one below that is the best fit for your research. If you are not sure, read the appropriate sections before making your selection.

☒ Life sciences ☐ Behavioural & social sciences ☐ Ecological, evolutionary & environmental sciences

For a reference copy of the document with all sections, see nature.com/documents/nr-reporting-summary-flat.pdf

Life sciences study design

All studies must disclose on these points even when the disclosure is negative.

Sample size	Sample sizes were calculated by performing a power analysis using data from previous vaccine experiments using similar adjuvants (alum and TLR7/8 agonists).
Data exclusions	No data were excluded from analyses.
Replication	Multiple independent experiments were run to generate cell line data. Multiple individual donors were used to generate primary human cell data. Individual male and female mice were used to generate anti-F1 antibody data and lead groups and controls were repeated. The murine challenge experiment was not repeated. Murine fentanyl challenge findings were supported by vaccination and challenge findings using the same vaccine (F1-CRM + alum + INI-4001) in rats and pigs as shown in a co-submitted manuscript (Crouse et al. Novel TLR7/8 agonist increases efficacy of vaccines against fentanyl misuse and overdose in rat and porcine models).
Randomization	Organisms were randomly organized into groups for mouse studies. Participants were randomly chosen from the blood donor pool to obtain primary cells used in this study.
Blinding	Investigators were not blinded for in vitro studies or murine vaccination studies. Data collected in these studies are objective measurements thus blinding is not necessary. Investigators were blinded for data collection and analysis in murine fentanyl challenge experiments.

Reporting for specific materials, systems and methods

We require information from authors about some types of materials, experimental systems and methods used in many studies. Here, indicate whether each material, system or method listed is relevant to your study. If you are not sure if a list item applies to your research, read the appropriate section before selecting a response.

Materials & experimental systems

n/a	Involved in the study	
☐	☒	Antibodies
☐	☒	Eukaryotic cell lines
☒	☐	Palaeontology and archaeology
☐	☒	Animals and other organisms
☒	☐	Clinical data
☒	☐	Dual use research of concern

Methods

n/a	Involved in the study	
☒	☐	ChIP-seq
☒	☐	Flow cytometry
☒	☐	MRI-based neuroimaging

Antibodies

Antibodies used	Antibodies used in this study were secondary antibodies for detection of mouse IgG, IgG1, and IgG2a. IgG: HRP labeled polyclonal goat anti-mouse IgG from Jackson ImmunoResearch or Southern Biotech. IgG1: HRP labeled polyclonal goat anti-mouse IgG1 from Alpha Diagnostic International. IgG2: HRP labeled polyclonal goat anti-mouse IgG2a from Alpha Diagnostic International
Validation	No primary antibodies were used in this study

Eukaryotic cell lines

Policy information about cell lines and Sex and Gender in Research

Cell line source(s)	All cell lines were purchased from Invivogen. HEK-Blue hTLR4, HEK-Blue hTLR7, and HEK-Blue hTLR8, along with HEK-Blue Null2 cells were used.
Authentication	None of the cell lines were authenticated
Mycoplasma contamination	None of the cell lines were recently tested for mycoplasma contamination
Commonly misidentified lines (See ICLAC register)	No commonly misidentified cell lines were used in this study.

Animals and other research organisms

Policy information about studies involving animals; ARRIVE guidelines recommended for reporting animal research, and Sex and Gender in Research

Laboratory animals	Mice, BALB/C, ages 7-16 weeks
Wild animals	No wild animals were used in this study
Reporting on sex	Only female mice were used in this study
Field-collected samples	Study did not use any field-collected samples
Ethics oversight	Mouse studies were approved by the University of Montana IACUC or the University of Minnesota IACUC

Note that full information on the approval of the study protocol must also be provided in the manuscript.
